# *FtMYB8* from Tartary buckwheat inhibits both anthocyanin/Proanthocyanidin accumulation and marginal Trichome initiation

**DOI:** 10.1186/s12870-019-1876-x

**Published:** 2019-06-18

**Authors:** Yunji Huang, Qi Wu, Shuang Wang, Jiaqi Shi, Qixin Dong, Panfeng Yao, Guannan Shi, Shuangxiu Xu, Renyu Deng, Chenglei Li, Hui Chen, Haixia Zhao

**Affiliations:** College of Life Science, Sichuan Agricultural University, No. 46, Xinkang Road, Ya’an, 625014 Sichuan Province China

**Keywords:** Anthocyanin, Arabidopsis, *FtMYB8*, Proanthocyanidin, Tartary buckwheat, Trichome

## Abstract

**Background:**

Because flavonoids and trichomes play crucial roles in plant defence, their formation requires fine transcriptional control by multiple transcription factor families. However, little is known regarding the mechanism of the R2R3-MYB transcription factors that regulate both flavonoid metabolism and trichome development.

**Results:**

Here, we identified a unique SG4-like-MYB TF from Tartary buckwheat, *FtMYB8*, which harbours the C2 repression motif and an additional TLLLFR repression motif. The expression profiles of *FtMYB8* combined with the transcriptional activity of *P*_*FtMYB8*_ promoter showed that *FtMYB8* mRNA mainly accumulated in roots during the true leaf stage and flowering stage and in bud trichomes and flowers, and the expression of this gene was markedly induced by MeJA, ABA and UV-B treatments but repressed by dark treatment. Overexpression of *FtMYB8* in Arabidopsis reduces the accumulation of anthocyanin/proanthocyanidin by specifically inhibiting *TT12* expression, which may depend on the interaction between FtMYB8 and TT8. Interestingly, this interaction may also negatively regulate the marginal trichome initiation in Arabidopsis leaves.

**Conclusions:**

Taken together, our results suggest that *FtMYB8* may fine-tune the accumulation of anthocyanin/proanthocyanidin in the roots and flowers of Tartary buckwheat by balancing the inductive effects of transcriptional activators, and probably regulate trichome distribution in the buds of Tartary buckwheat.

**Electronic supplementary material:**

The online version of this article (10.1186/s12870-019-1876-x) contains supplementary material, which is available to authorized users.

## Background

Flavonoids, including anthocyanins, PAs and rutin, which are bioactive products of the phenylpropanoid pathway, have extensive functions in plants, such as in the resistance to pathogen attacks, herbivorous defence, protection from UV damage and attraction of insects for pollination and seed dispersal [1]. Generally, the transcription of enzyme-encoding genes in the flavonoid pathway is regulated by multiple TFs, which mainly include WD40s, bHLHs and MYBs [2]. Among these TFs, the biological function of MYB proteins is complex and has been extensively studied.

The R2R3-MYB TF sub-family, which is deemed the largest sub-family of MYB TFs in *Arabidopsis thaliana*, has been classified into 23 sub-groups [3]. R2R3-MYB TF can participate as a separate transcriptional regulator in the flavonoid pathway. Among the R2R3-MYB TFs identified thus far in Arabidopsis, only members of SG4, including AtMYB32, AtMYB7, AtMYB4 and AtMYB3, function as separate transcription repressors in the phenylpropanoid pathway [3–6]. Notably, the MYB protein can also interact with bHLH and WD40 together to form a MBW ternary complex that is indispensable for the biosynthesis of anthocyanin/PA and trichome cell patterning [7]. In Arabidopsis, the PAP1/PAP2/MYB113/MYB114-GL3/EGL3/TT8-TTG1 complex positively regulates anthocyanin biosynthesis [8]; the TT2-TT8-TTG1 complex is essential for PA accumulation in the seed coat [9]; and the GL1-GL3/EGL3-TTG1 complex is a key determinant of epidermal cell fate in shoots [10].

Even more remarkably, some MYB TFs identified to date, including R3-MYB and R2R3-MYB, function as co-repressors in anthocyanin and/or PA synthesis and/or epidermis cell fate determination by competitively binding to the bHLH protein. CPC, TRY and ETC1, which repress trichome cell fate determination, block the formation of the GL1-GL3/EGL3-TTG1 complex by competitively binding to the GL3/EGL3 protein in Arabidopsis [11–13]; AtMYBL2 and PhMYBx (*Petunia hybrida*) function as repressors in anthocyanin/PA synthesis via competitive binding to the bHLH protein [14, 15]; Interestingly, when *PtrRML1* (*Populus trichocarpa*) is expressed in Arabidopsis, PtrRML1 negatively regulates both flavonoid biosynthesis and epidermal cell fate in transgenic Arabidopsis by interacting with AtGL3 [16]. These studies have shown that flavonoid metabolism and trichome development can be regulated individually or simultaneously by some members of R3-MYB family. However, among the R2R3-MYB TFs identified to date, only *FaMYB1* (*Fragaria ananassa*), *PhMYB27* (*Petunia hybrida*), *VvMYBC2-L3* and *VvMYBC2-L1* (*Vitis vinifera*) function as the co-repressors of anthocyanin/PA synthesis by competitive binding to bHLH protein [15, 17, 18]. These R2R3-MYB TFs with co-inhibitory effects share a conserved C2 motif (pdLNLD/LLxiG/S), which converts transcriptional activators to strong suppressors [3, 19], and these TFs have more complex combinations of motifs than SG4-MYBs. However, R2R3-MYB, which not only inhibits flavonoid synthesis but also regulates trichome development, remains poorly understood. Considering that *PtrRML1* negatively regulates both flavonoid biosynthesis and trichome development in transgenic Arabidopsis when *PtrRML1* is expressed in Arabidopsis, we hypothesize that some unique R2R3-MYBs with co-inhibitory effects may also be involved in trichome development.

As a crop in mountainous areas, Tartary buckwheat (*Fagopyrum tataricum*) exhibits barren soil tolerance, stress tolerance and high nutrient content [20]. In recent years, research interest in Tartary buckwheat has increased because this crop contains a variety of functional flavonoids, including anthocyanins, PAs and rutin [21]. Notably, little is known regarding the potential regulatory mechanism that leads to rutin being the major flavonoid in Tartary buckwheat, and not anthocyanins or PAs. This phenomenon may be attributed to the complex and refined regulatory network, which is composed of numerous TFs in the flavonoid-related pathway of Tartary buckwheat [22]. For instance, this crop possesses a large number of MYB TFs that are thought to regulate mainly the biosynthesis of anthocyanins/PAs, including some transcriptional activators (six SG5-MYBs and three SG6-MYBs) and some transcriptional repressors (five SG4-MYBs and four SG4-like-MYBs). Most noticeably, SG4-like-MYBs, which are thought to have played diverse roles in the regulation of flavonoid metabolism over the course of the evolution of these proteins, are almost non-existent in other plants. To date, in Tartary buckwheat, only *FtMYB15*, which is an SG4-like-MYB, plays a positive role in the biosynthesis of anthocyanin/PA in Arabidopsis [23]. The function of FtMYB15 is slightly inconsistent with it structure, given that this protein possesses a C2 inhibitory motif, which also indicates that SG4-like MYBs have evolved to play diverse roles. Thus, to elucidate the mechanism underlying the regulation of flavonoid metabolism, it is necessary to identify other *SG4-like-MYBs* in Tartary buckwheat. In this study, our results indicate that *FtMYB8*, the first unique R2R3-MYB TF gene identified to date, inhibits both anthocyanin/PA accumulation and marginal trichome initiation at specific developmental stages and in response to hormone signals and environmental factors.

## Results

### Expression analysis and validation of *SG4-like-MYBs*

**Fig. 1:**
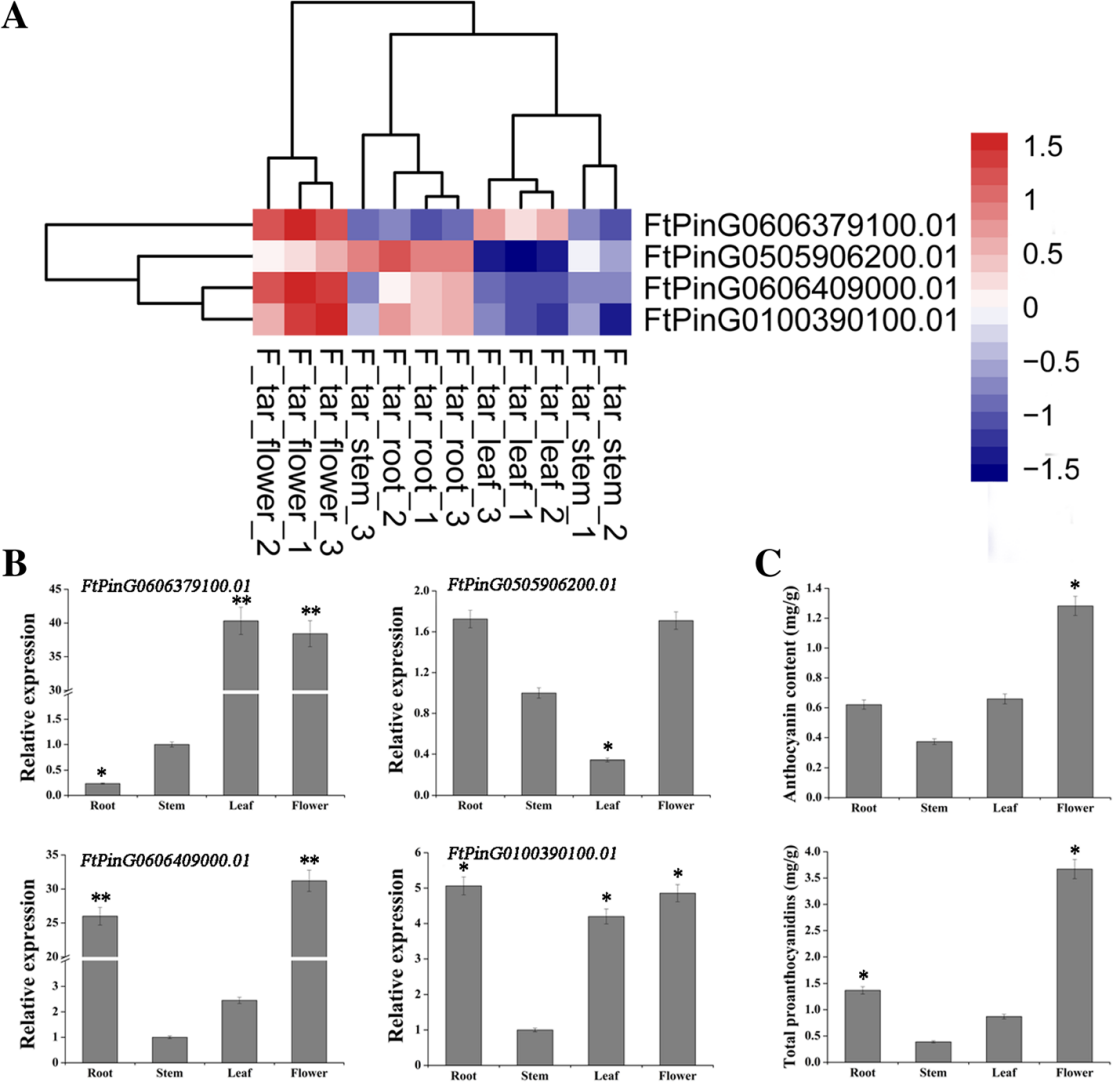
Analysis of *SG4-like-MYB* expression and anthocyanin/PA content at the flowering stage. **a** Heat map of *SG4-like-MYBs*. Each column represents one tissue, and each line represents one gene that is displayed on the right. The colour intensity of the blue and red rectangles reflect low and high Z-scores for mRNA accumulation. **b** Expression pattern of *SG4-like-MYBs*. *FtH3* was used as a reference gene. The accumulation of each *SG4-like-MYB* mRNA in the stems was defined as “1” for each developmental stage. Means were calculated from three repeats, and error bars reflect ±SDs. **c** Anthocyanin/PA content in different tissues

Based on the genomic database [24] and our transcriptomic database (data not shown), *SG4-like-MYBs* were identified in Tartary buckwheat. Both the *FtPinG0606379100.01* and *FtPinG0606409000.01* genes are located on the 5th chromosome, and the *FtPinG0505906200.01* and *FtPinG0100390100.01* genes are located on the 7th chromosome and 4th chromosome, respectively. Then, the expression levels of the genes were visualized as a heat map generated by pheatmap 1.0.10 (Fig. 1a). High accumulation of all *SG4-like-MYB* mRNAs was observed in the flowers and roots, except for *FtPinG0606379100.01* mRNA, which exhibited high abundance in the flowers and leaves; relatively low accumulation of all *SG4-like-MYB* mRNAs was detected in the leaves and stems, except for *FtPinG0606379100.01* mRNA, which exhibited low abundance in the stems and roots. Then, the transcriptional levels of the *SG4-like-MYBs* were validated by qRT-PCR, and the results were consistent with the transcriptomic data (Fig. 1b). Furthermore, the *FtPinG0606379100.01* and *FtPinG0606409000.01* genes were highly expressed in flowers/leaves and in flowers/roots, respectively, clearly demonstrating tissue specificity. Subsequently, we determined the levels of anthocyanins/PAs in various tissues. As shown in Fig. 1c, the highest anthocyanins/PA content was observed in the flowers, followed by the roots, leaves and stems, demonstrating positive correlations with the *FtPinG0606409000.01* expression profile. Therefore, we speculated that this gene may positively regulate anthocyanin/PA biosynthesis in Tartary buckwheat. *FtPinG0606409000.01* was selected for further experiments and named *FtMYB8*.

### Isolation and identification of *FtMYB8*

*FtMYB8* possesses a 729 bp CDS and encodes an MYB protein with 242 amino acids, and the length of the gDNA of this gene is 1153 bp (GenBank accession no. MK128409). Our analyses suggested that the gDNA of this gene is composed of two introns (131–308 and 439–684 bp) and three exons (1–130, 309–438 and 684–1153 bp).

**Fig. 2:**
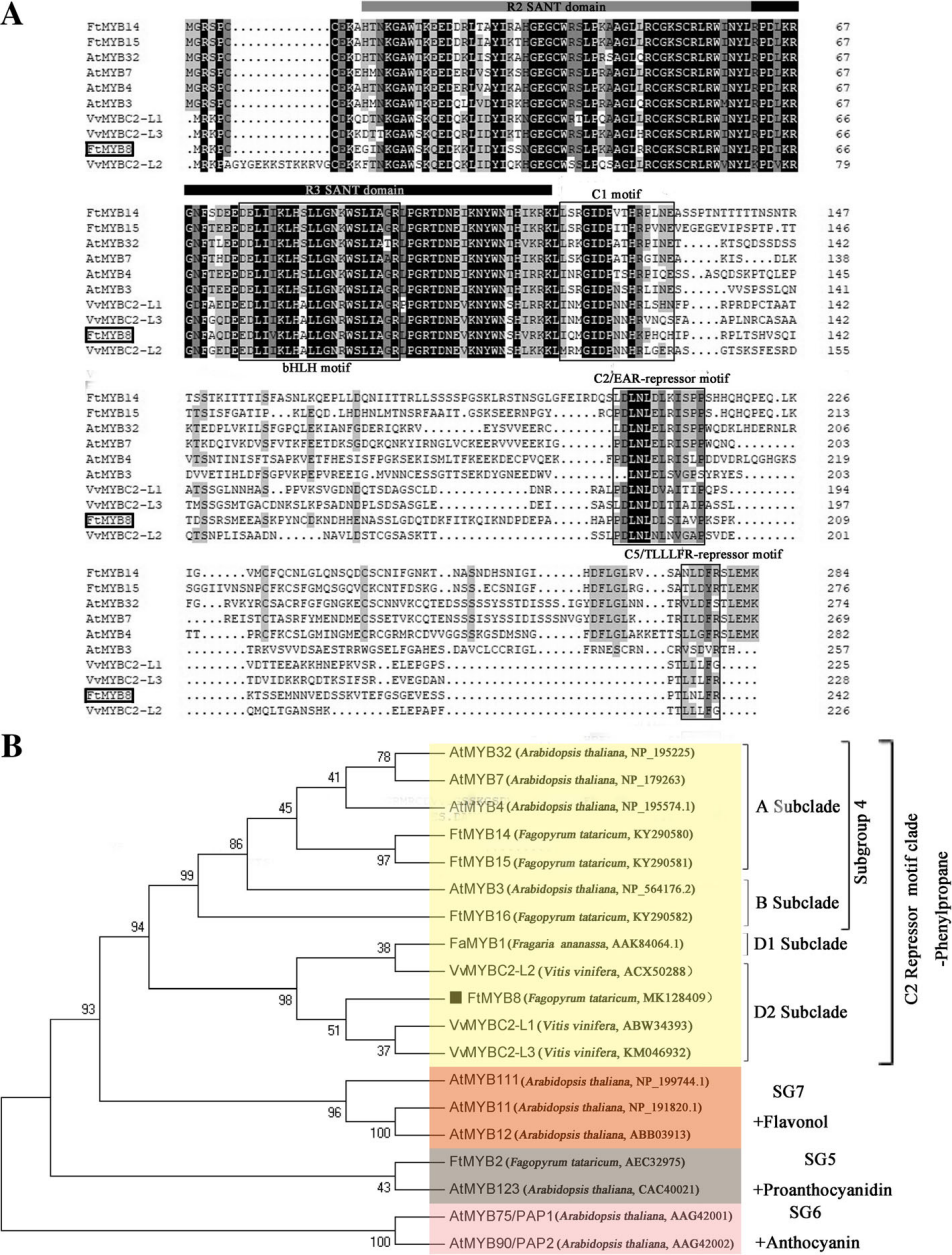
Molecular identification of FtMYB8. **a** Multiple sequence alignment of FtMYB8. The R2 and R3 SANT domains are underlined. The bHLH interaction motif and three conserved motifs are indicated with black boxes. **b** Phylogenetic relationships of FtMYB8. The GenBank accession numbers are displayed to the right of the protein names. FtMYB8 is highlighted with a black square

Subsequently, the multiple sequence alignment of FtMYB8 and other SG4-MYBs was performed to further identify conserved motifs. The analysis results indicated that FtMYB8 has two MYB repeats at the N terminus. Moreover, a bHLH motif ([D/E]Lx_2_[R/K]x_3_Lx_6_Lx_3_R) was identified in the R3 region [25]; two conserved motifs (C1 motif and C2 motif) of SG4-MYB were identified [3]; and a novel repression motif (C5 motif) was identified at the C terminus [19] (Fig. 2a). In addition, phylogenetic analysis indicated that FtMYB8 was grouped with subclade D2 of the R2R3-MYB C2 repressor, which functions as a suppressor in both PA and anthocyanin biosynthesis [18]. Thus, FtMYB8 has a potential inhibitory function in flavonoid biosynthesis (Fig. 2b).

### Spatiotemporal expression of *FtMYB8* in Tartary buckwheat

*FtMYB8* expression was monitored in the roots, stems, leaves and buds of Tartary buckwheat at different developmental stages by qRT-PCR (Additional file 1: Figure S1). The highest abundance of FtMYB8 mRNA was detected in the buds at each growth stage, followed by the roots. The expression of *FtMYB8* in both stems and leaves was low at all developmental stages. Taken together, our results show that *FtMYB8* was expressed mainly in roots and buds, and the expression of this gene was low in both stems and leaves.

### Expression of *FtMYB8* in Tartary buckwheat under stress treatments

To better understand the response of *FtMYB8* to environmental factors and hormones, the expression level of *FtMYB8* was monitored under dark, UV-B, ABA and MeJA treatments. As shown in Additional file 1: Figure S2, under normal conditions and dark treatment, no marked change was observed in *FtMYB8* transcriptional levels within 16 h. Moreover, after UV-B treatment, the *FtMYB8* mRNA accumulated rapidly and peaked at 3 h, after which, the levels decreased but remained significant. In addition, ABA and MeJA treatments induced the expression of *FtMYB8* within 0.5 h, and then, the abundance of *FtMYB8* mRNA decreased rapidly and then remained relatively stable.

### Identification and functional analysis of *P*_*FtMYB8*_

The 5′ upstream sequence of the *FtMYB8* gene was determined from the genomic data of Tartary buckwheat and preliminarily analysed with the PlantCARE database. Then, a 2313 bp promoter was cloned and identified by sequencing (Additional file 1: Table S1). Analysis results showed that there were several cis-regulatory elements in *P*_*FtMYB8*_ that were putatively involved in environmental response and hormone response in plants (Additional file 1: Table S2). Cis-acting elements involved in environmental response include the GA motif, GATT motif, and GT1 motif, all of which are light-responsive elements. Cis-acting elements involved in hormone response include ABRE (involved in ABA responsiveness), the CGTCA motif and the TGACG motif (involved in MeJA responsiveness). These results suggest that the activity of the *P*_*FtMYB8*_ promoter may be affected by light, ABA and MeJA.

**Fig. 3:**
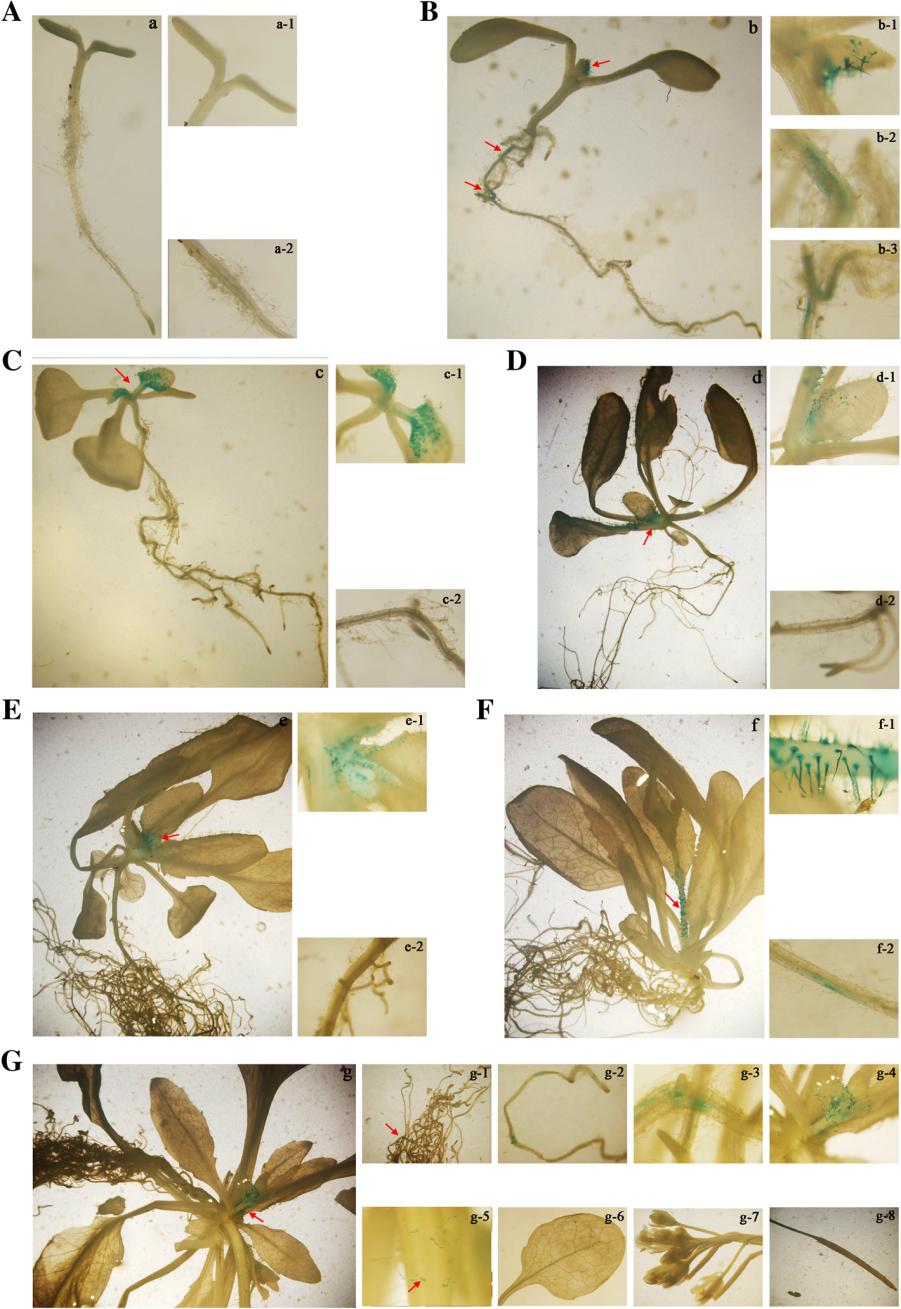
Histochemical localization of *GUS* expression under *P*_*FtMYB8*_ promoter control in transgenic plants. **a**-**g** Histochemical staining of 4-, 6-, 11-, 18-, 25-, 32- and 42-day-old *P*_*FtMYB8*_*:GUS* seedlings. **a**-**g** Histochemical GUS staining of whole seedlings. (a-1) Cotyledons and hypocotyl of a 4-day-old plant. (a-2) Root of a 4-day-old plant. (b-1) Bud of a 6-day-old plant. (b-2)-(b-3) Roots of a 6-day-old plant. (c-1) Bud of an 11-day-old plant. (c-2) Root of an 11-day-old plant. (d-1) Bud of an 18-day-old plant. (d-2) Root of an 18-day-old plant. (e-1) Bud of a 25-day-old plant. (e-2) Root of a 25-day-old plant. (f-1) Stem of a 32-day-old plant. (f-2) Root primordia of a 32-day-old plant. (g-1)-(g-3) Root primordia of a 42-day-old plant. (g-4) Crowded bud of a 42-day-old plant. (g-5) Stem of a 42-day-old plant. (g-6) Leaf of a 42-day-old plant. (g-7) Flower of a 42-day-old plant. (g-8) Silique of a 42-day-old plant

Moreover, our qRT-PCR results indicate that there was a relatively high abundance of *FtMYB8* mRNA in the roots, buds and flowers of Tartary buckwheat during the growth and development process. It is widely recognized that promoters are key for the regulation of gene expression. To gain insight into the potential regulatory mechanism of *FtMYB8* expression, *P*_*FtMYB8*_ was cloned and ligated into the pBI101:GUS vector. Transgenic Arabidopsis harbouring *P*_*FtMYB8*_ was generated and verified by PCR. Then, the GUS activity was investigated by GUS staining at seven different developmental stages of transgenic Arabidopsis (Fig. 3). There was no GUS activity in 4-day-old plants (Fig. 3a). In 6-day-old plants, GUS activity was observed in the bud trichomes and roots (Fig. 3b). In 11-, 18- and 25-day-old plants, GUS activity was observed in only the bud trichomes (Fig. 3c, e). In 32-day-old plants, GUS activity was observed in the stem trichomes and root primordia (Fig. 3f). In 42-day-old plants, GUS activity was observed in the root primordia, crowded bud trichomes and stem trichomes, not in the leaves, flowers and siliques (Fig. 3g). Overall, the *P*_*FtMYB8*_ promoter has obvious transcriptional activity in the roots at the true leaf stage, root primordia and stem trichomes at the flowering stage and all bud trichomes.

**Fig. 4:**
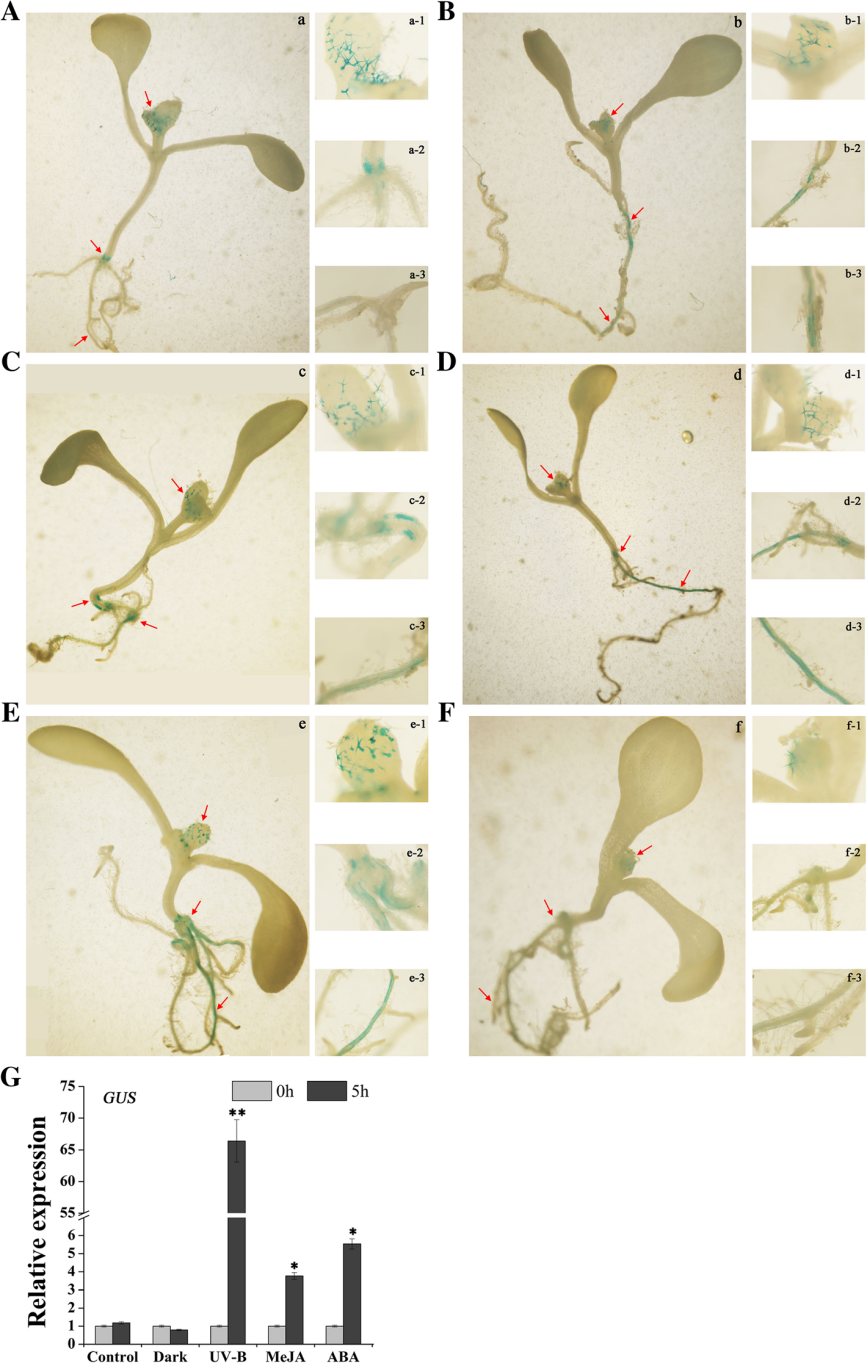
Analyses of the response of the *P*_*FtMYB8*_ promoter to environmental factors and hormone signals. **a**-**f** Histochemical GUS staining of *P*_*FtMYB8*_*:GUS* seedlings under normal conditions (**a**, **b**) or subjected for 5 h to 50 μM MeJA (C), 10 μM ABA (D), UV-B (**e**) or dark (**f**). (a-f) Histochemical GUS staining of whole seedlings. (n-1)-(n-3) Histochemical GUS staining of the buds (n-1), roots (n-2) or roots (n-3), where n represents a-f. **g** Abundance analyses of *GUS* mRNA in WT and transgenic Arabidopsis whole seedlings. Total RNA was extracted after 5 h of treatment, and each sample contained at least 30 seedlings. Relative expression of the GUS gene was evaluated by the 2^-ΔΔCT^ method, and the accumulation of *GUS* mRNA in whole transgenic seedlings under normal conditions was defined as “1”. Arabidopsis *Atactin2* was used as a reference gene. Means were calculated from three repeats, and error bars reflect ±SDs. ** and * represent extremely significant differences and significant differences, respectively (*P < 0.05, **P < 0.01)

To study the response of the *P*_*FtMYB8*_ promoter to environmental factors and hormone signals, UV-B, dark, MeJA and ABA treatments were performed (Fig. 4). As shown in Fig. 4a and b, under normal conditions, there was no significant change in GUS activity in the bud trichomes and roots of the seedlings within 5 h. In addition, no marked change was observed in *GUS* transcriptional levels within 5 h (*P* > 0.05) (Fig. 4g). Under MeJA treatment, there was no obvious difference in GUS activity in the bud trichomes of the seedlings within 5 h; nevertheless, enhance of GUS activity was observed in the roots of the seedlings within 5 h (Fig. 4c), and a significant increase was also observed in the abundance of *GUS* mRNA within 5 h (*P* < 0.05) (Fig. 4g). The effect of ABA treatment on the transcriptional activity of the *P*_*FtMYB8*_ promoter was similar to that of MeJA treatment (Fig. 4d and g). After UV-B treatment for 5 h, significant enhance of the GUS activity was observed in the roots of the seedlings, not in the bud trichomes of the seedlings (Fig. 4e). Simultaneously, the abundance of *GUS* mRNA exhibited an extremely significant increase (*P* < 0.01) (Fig. 4g). After dark treatment for 5 h, the GUS activity decreased slightly in the roots and bud trichomes of the seedlings (Fig. 4f); In addition, no marked decrease was observed in *GUS* transcriptional levels (P > 0.05) (Fig. 4g). Taken together, our results show that the transcriptional activity of the *P*_*FtMYB8*_ promoter increased significantly under MeJA, ABA and UV-B treatments and was repressed by dark treatment, but not significantly.

### FtMYB8 has no individual transcriptional activity

For investigating the transcriptional activity of FtMYB8, the yeast one-hybrid assay was operated in this study. As shown in Additional file 1: Figure S3A and B, like the empty control (NC1), AH109 cells harbouring the pBridge-*FtMYB8* plasmid (F8) grew well on the SD/−Trp medium but not on SD/−Trp/−His, which indicated FtMYB8 could not activate the expression of *His* in yeast. Moreover, there was no change in colour of AH109 cells with pBridge-*FtMYB8* after the reaction with X-gal (Additional file 1: Figure S3C). However, AH109 cells including pBridge-*FtbHLH2* exhibited the blue colouration. These results suggested that the FtMYB8 also could not activate the reporter gene *LacZ*. Taken together, all of these results demonstrated that FtMYB8 has no individual transcriptional activity in yeast.

### Overexpession of *FtMYB8* decreases the anthocyanin/PA levels in transgenic tobacco plants

**Fig. 5:**
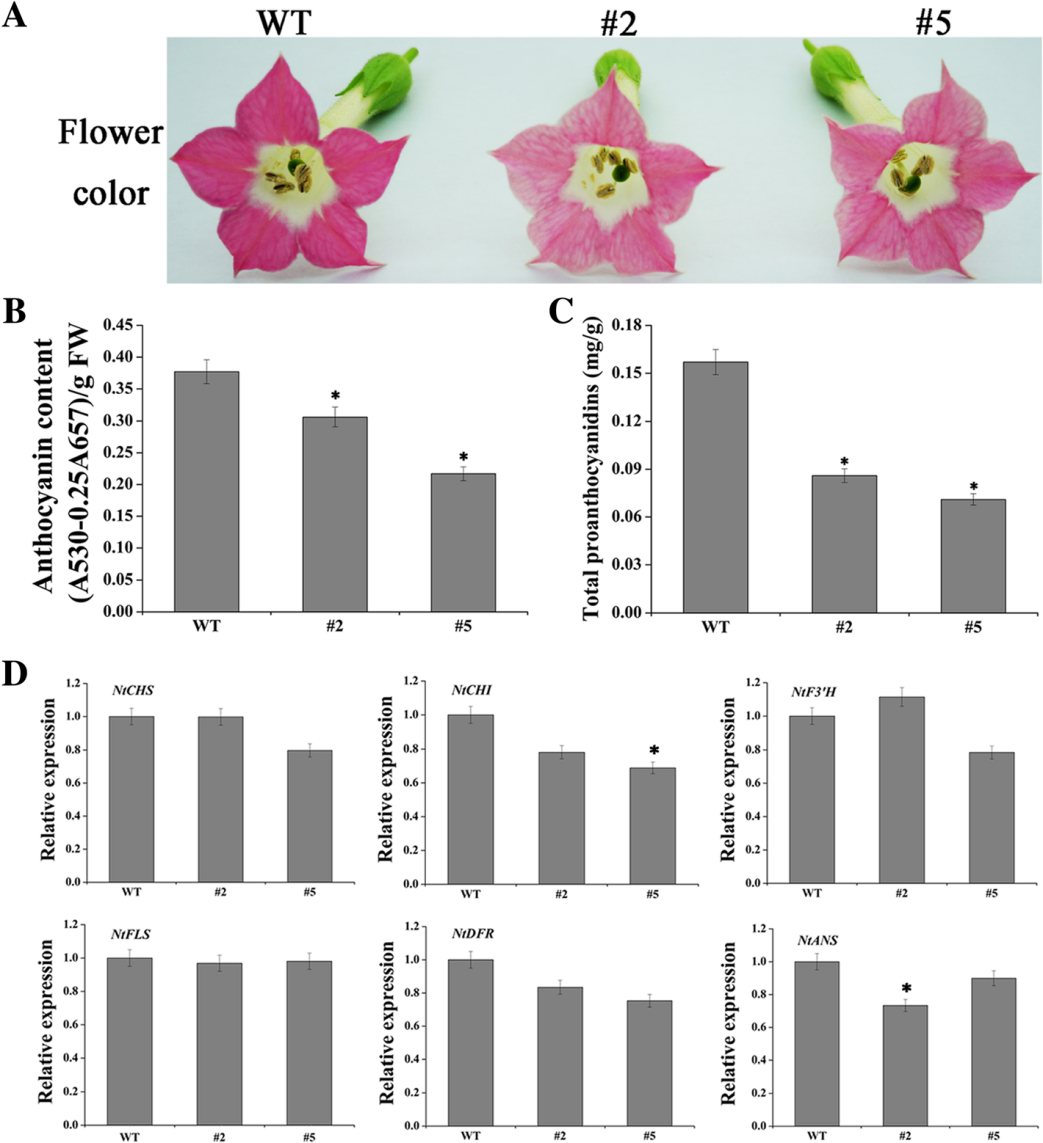
Overexpression of *FtMYB8* reduces the anthocyanin/PA content in transgenic tobacco plants. **a** Changes in the floral phenotype in tobacco plants overexpressing *FtMYB8*. **b**, **c** Anthocyanins/PA content in transgenic tobacco leaves. **d** Detection of flavonoid biosynthetic gene transcriptional levels in *FtMYB8* transgenic tobacco leaves. mRNA accumulation of six genes was monitored by qRT-PCR in the WT and transgenic lines. Relative expression was evaluated by the 2^−ΔΔCT^ method, and gene expression levels were defined as “1” in WT tobacco. Tobacco *Ntβ-actin* was used as a reference gene. Means were calculated from three repeats, and error bars reflect ±SDs. *P < 0.05

To directly observe the effect of *FtMYB8* on flavonoid biosynthesis, *FtMYB8*-overexpressing lines of tobacco (#2 and #5) were generated and verified by qRT-PCR (Additional file 1: Figure S4). As shown in Fig. 5a**,** the flowers of WT tobacco show red colouration. However, compared with WT tobacco, the *FtMYB8*-overexpressing tobacco lines show significantly lighter petal pigmentation. These results indicated that anthocyanin accumulation in the petals of the *FtMYB8*-overexpressing tobacco lines was likely lower than that in WT tobacco petals. To confirm this hypothesis, total anthocyanin/PA levels were determined. The results suggested that the anthocyanin/PA content of the overexpression lines was significantly lower than that of WT tobacco (*P* < 0.05) (Fig. 5b).

To elucidate the regulatory mechanism of *FtMYB8* in the flavonoid biosynthetic pathway, the transcriptional levels of the flavonoid biosynthetic genes were monitored (Fig. 5c). The expression levels of *NtFLS* and *EBGs*, including *NtCHS*, *NtCHI* and *NtF3’H*, almost showed no significant change between the transgenic tobacco plants and WT plants. Surprisingly, the expression levels of *LBGs*, including *NtDFR* and *NtANS*, also showed no marked difference between the transgenic tobacco plants and WT plants (*P* > 0.05). These results could not sufficiently explain why the total anthocyanin/PA content decreased in the transgenic tobacco plants.

### FtMYB8 inhibits anthocyanin/PA accumulation and marginal trichome initiation in transgenic Arabidopsis plants

To further ascertain the function of *FtMYB8*, *FtMYB8* overexpression in Arabidopsis (#1, #3 and #4) was carried out and verified by qRT-PCR (Additional file 1: Figure S5). Then, to study the subcellular localization of the FtMYB8 protein, the roots of *FtMYB8*-*YFP* transgenic Arabidopsis plants were examined by confocal microscopy (Additional file 1: Figure S6). The blue marker (nuclear marker) and FtMYB8-YFP protein were localized at the same location. These results suggested that FtMYB8 could be a nuclear protein.

**Fig. 6:**
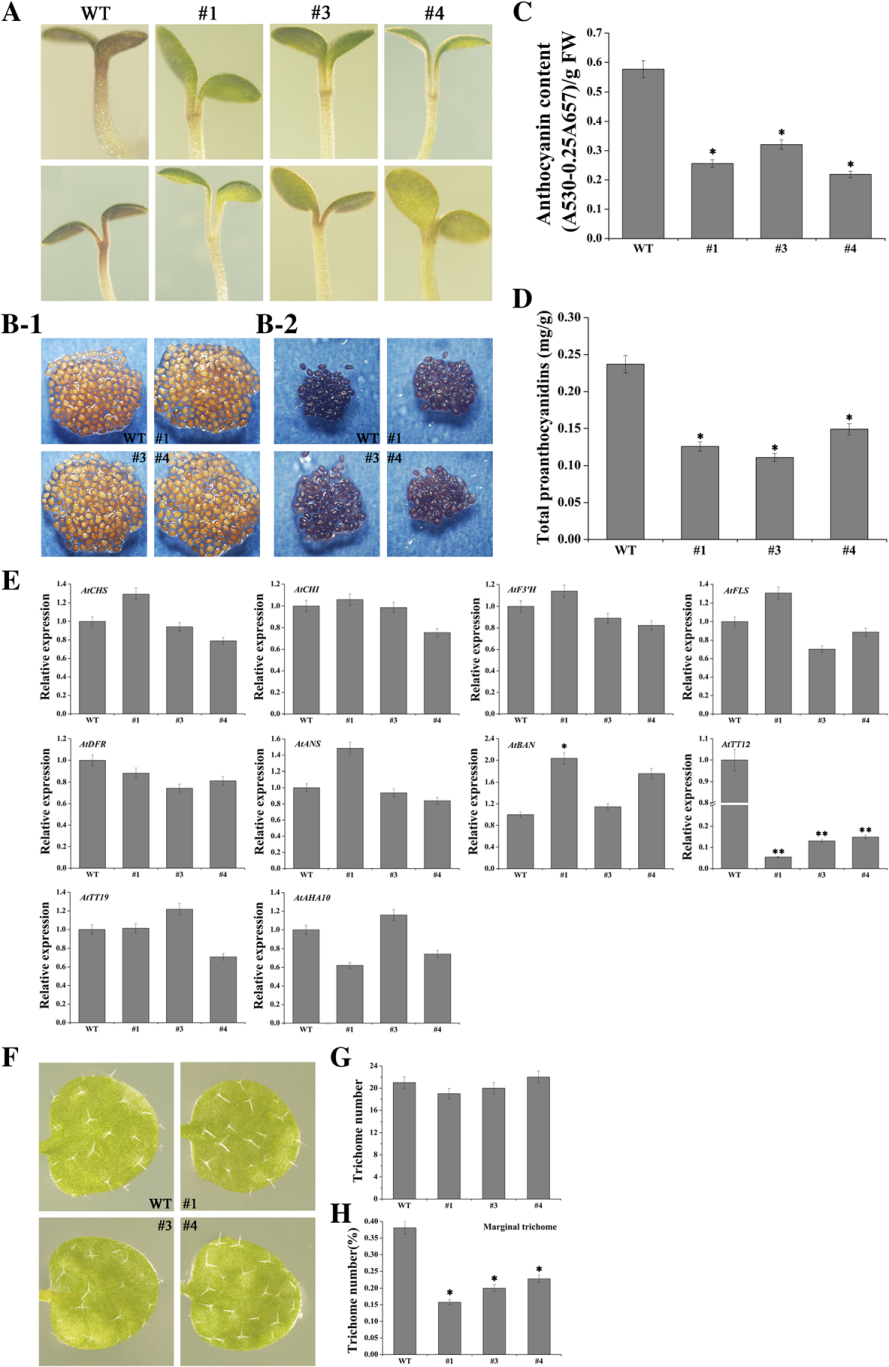
Effect of *FtMYB8* on the accumulation of anthocyanin/PA, gene expression and development of marginal trichomes. **a** Photographs of WT and transgenic seedlings grown in MS medium with 3% sucrose. (B-1) Mature Arabidopsis seeds. (B-2) PA accumulation in mature seeds stained by vanillin-HCl. **c**,**d** Anthocyanin/PA content in transgenic lines and WT plants. **e** Each sample used for total RNA extraction contained at least 50 seedlings. The mRNA abundances of ten genes were monitored by qRT-PCR in the WT and transgenic lines. The relative expression was evaluated by the 2^−ΔΔCT^ method, and gene expression levels were defined as “1” in WT Arabidopsis seedlings. Arabidopsis *Atactin2* was used as a reference gene. **f** Comparison of trichome distribution between transgenic plants and WT plants at the true leaf stage by stereomicroscopy. **g** Total number of trichomes at the true leaf stage in the WT and transgenic lines. Each sample used to count the total number of trichomes contained at least 15 leaves. **h** Percentage of marginal trichomes in true leaves in the WT and transgenic lines. Each sample used to calculate the percentage of marginal trichomes contained at least 15 leaves. Means were calculated from three repeats and error bars reflect ±SDs. *P < 0.05, **P < 0.01

First, reduced seed dormancy was observed in transgenic Arabidopsis seeds, as indicated by the early germination compared to WT seeds (Additional file 1: Figure S7). Moreover, anthocyanin accumulation in Arabidopsis seedlings on MS medium with 3% sucrose was observed (Fig. 6a). These results suggested that pigment deposition in WT Arabidopsis seedlings was greater than that in *FtMYB8*-*YFP* transgenic seedlings (Fig. 6B-1). In addition, the seed coats of WT Arabidopsis exhibited deeper pigmentation than those of the transgenic lines, and after the vanillin-HCl staining assay, the intensity of seed coat colouration increased (Fig. 6B-2). Normally, a darker colour indicates greater PA accumulation, as per the principle of the staining assay [26]. These results indicated reduced PA accumulation in the *FtMYB8*-*YFP* transgenic Arabidopsis seed coats compared to the WT Arabidopsis seed coats. Then, the PA and anthocyanin levels were measured. The results showed that the levels of both anthocyanin and PA were significantly decreased in the transgenic plants compared to the WT plants (Fig. 6c and d).

To identify the regulatory mechanism of *FtMYB8* in PA and anthocyanin accumulation, the transcriptional levels of *EBGs*, *LBGs* and *TGGs* were monitored in Arabidopsis. As shown in Fig. 6e, the expression levels of *EBGs*, *LBGs* and *AtFLS* showed almost no significant difference between the transgenic Arabidopsis plants and WT plants. *TGGs*, which encode H^+^-ATPase, glutathione-S-transferase and the MATE transporter, have been shown to be involved in anthocyanin and/or PA oxidation, transport and modification in Arabidopsis [27–29]. Thus, we speculated that FtMYB8 is involved in the transcriptional regulation of this group of enzyme-encoding genes. The *AtTT19* and *AtAHA10* expression levels also no marked change between transgenic Arabidopsis plants and WT plants (*P* > 0.05). Interestingly, the *AtTT12* transcriptional levels were significantly downregulated in the transgenic lines compared to WT Arabidopsis (*P* < 0.01). In general, *FtMYB8* could decrease anthocyanin/PA accumulation.

Considering that the *P*_*FtMYB8*_ promoter has obvious transcriptional activity in bud trichomes, we speculated that FtMYB8 may be involved in trichome development in young leaves. Interestingly, we observed that the distribution of trichomes, rather than the number and morphogenesis of trichomes, was significantly different in true leaves between the WT and transgenic lines (Fig. 6f and g). The percentage of marginal trichomes in the true leaves of WT plants was more than that of transgenic plants, which indicated that *FtMYB8* is important for marginal trichome initiation (Fig. 6h).

### FtMYB8 interacts with AtTT8/FtTT8/FtGL3 in yeast

Our results suggested that FtMYB8 has no individual transcriptional activity in yeast; however, FtMYB8 could reduce the expression level of *AtTT12* in Arabidopsis. These results indicated that FtMYB8 has transcriptional activity in Arabidopsis that may depend on the interacting proteins [30]. It has been established that the transcriptional activity of some MYBs were dependent on forming MBW complexes by interacting with bHLHs [31]. Considering that FtMYB8 contains the bHLH-interacting motif in the R3 domain (Fig. 2a), we speculated that FtMYB8 inhibits the accumulation of anthocyanin/PA in Arabidopsis by interacting with bHLHs. A yeast two-hybrid system was used to confirm this hypothesis.

**Fig. 7:**
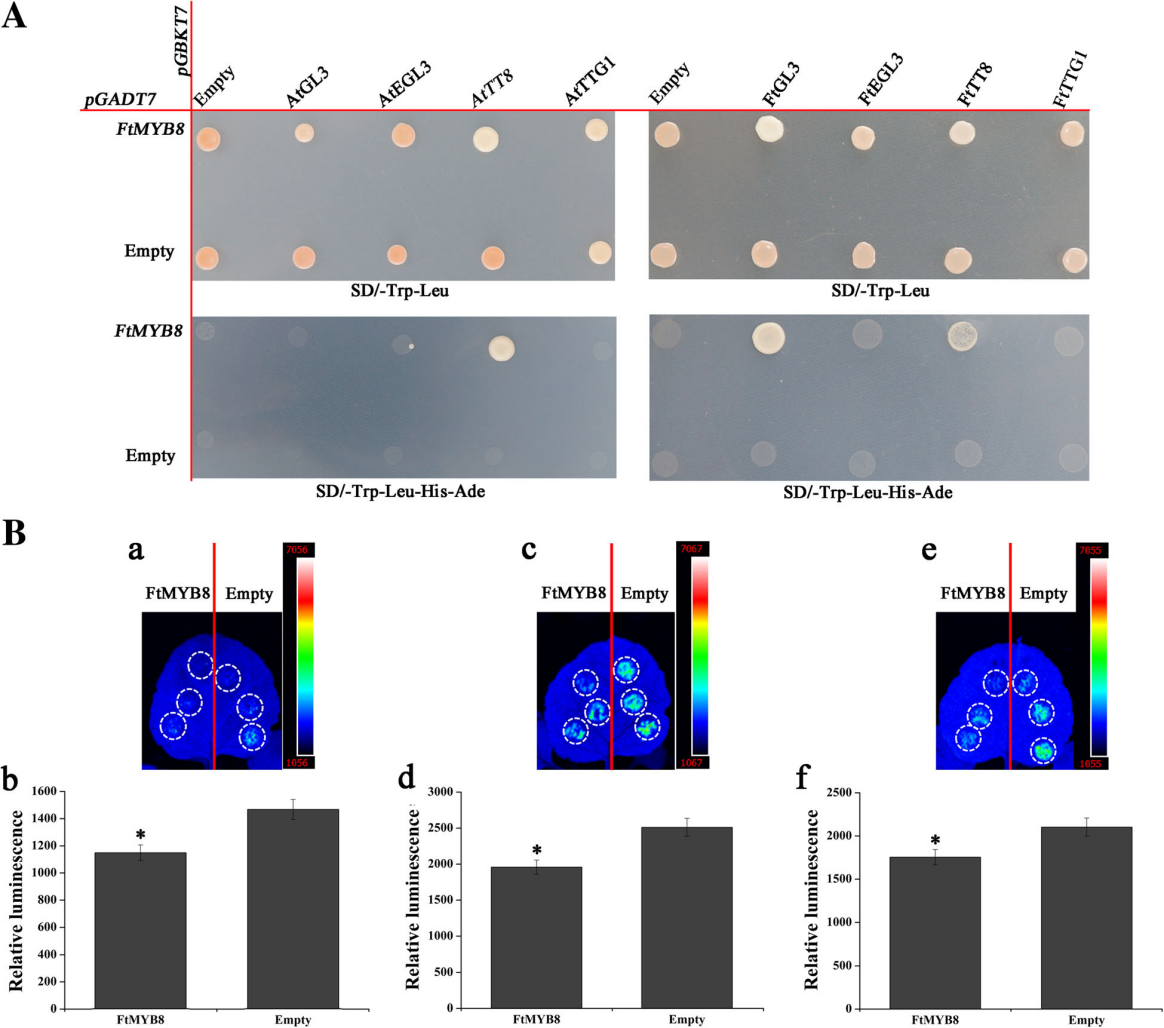
Molecular interaction identification of FtMY8 transcription factor. **a** FtMYB8 interacts with AtTT8/FtTT8/FtGL3 in yeast. Image showing the growth of AH109 [*pGADT7*-*FtMYB8* × *pGBKT7*-*AtTT8*/−*AtGL3*/−*AtEGL3*/−*AtTTG1*/−*FtTT8*/−*FtGL3*/−*FtEGL3*/−*FtTTG1*] yeast on SD/−Leu-Trp plates and SD/−Leu-Trp-His-Ade plates. **b** Transient expression assays displaying that FtMYB8 inhibits *AtTT12* expression. (a, c and e) Representative images of *Nicotiana benthamiana* leaves 48 h after infiltration are shown. (b, d and f) Quantitative analysis of luminescence intensity represent a, c and e, respectively. Means were calculated from three repeats and error bars reflect ±SDs. *P < 0.05

As shown in Fig. 7a, the yeast cells possessing all the bait and prey combinations were grew well on SD/−Leu-Trp medium. Nevertheless, white colonies was observed in the yeast which possessing both pGADT7-*FtMYB8* and pGBKT7-*AtTT8/FtTT8/FtGL3* on SD/−Leu-Trp-His-Ade plates, suggesting the reporter gene of *ADE2* and *HIS3* were activated. All of these results suggested that FtMYB8 strongly interacted with AtTT8 and FtGL3; FtMYB8 also interacted with FtTT8, but the strength of its interactions seemed weak.

### FtMYB8 inhibits the expression of *AtTT12*

To further confirm that FtMYB8 could inhibits the expression of *AtTT12*, we have performed a transient assay to detect the effect of FtMYB8 on the expression of *P*_*AtTT12*_:*Luc* reporter containing the *AtTT12* promoter fragments fused with *LUC* gene. As shown in Fig. 7b, FtMYB8 decreased significantly the luminescence intensity of *P*_*AtTT12*_:*Luc* compared with the empty control (Fig. 7B-a, −c and -e). These results were consistent with the quantitative measurement of fluorescence intensity (Fig. 7B-b, −d and -f).

## Discussion

It has been widely reported that MBW ternary complexes are indispensable for biosynthesis and accumulation of anthocyanin/PA and trichome initiation in plants [32]. However, the members of the MBW complex and the associated regulatory mechanism remain elusive. In this paper, *FtMYB8*, a unique SG4-like-MYB TF from Tartary buckwheat, was isolated and identified. Further analysis showed that FtMYB8 possessed C-terminal C1, C2 and C5 motifs and was clustered to the D2 subclade of R2R3-MYB C2 repressors with VvMYBC2-L1 and VvMYBC2-L3 [18]. It has been proven that VvMYBC2-L1 and VvMYBC2-L3 may balance the inductive effects of transcriptional activators by interacting with bHLH TFs [18]. Moreover, a potential characteristic motif for bHLH interaction was discovered in the R3 domain of this protein, and FtMYB8 exhibited no individual transcriptional activity. These results allow us to hypothesize that *FtMYB8* also exhibits repression of flavonoid biosynthesis, which may depend on the interacting proteins.

In this study, compared to WT plants, reduced pigmentation was observed in the flowers of transgenic tobacco plants and seedlings of transgenic Arabidopsis plants, and reduced PA accumulation was observed in the seed coats of transgenic Arabidopsis plants. Previous studies have shown that MYBs function as regulators in the flavonoid pathway by activating or repressing the expression of *EBGs* and *LBGs* [33]. However, the mRNA abundances of *EBGs* and *LBGs* were not significantly different between WT and transgenic seedlings. Interestingly, the expression of *AtTT12*, which encodes a proton antiporter that is responsible for transporting anthocyanin/PA precursors into the vacuole, was significantly downregulated in transgenic seedlings [29]. It has been established that *tt12* mutant seeds exhibited pale brown seed coats and reduced seed dormancy compared to WT seed (these phenotypes were curated by the Arabidopsis Biological Resource Center). Consistent with these results, the seeds of the transgenic lines also exhibited early germination (Additional file 1: Figure S7). These results have to convince us a potential mechanism that FtMYB8 is involved in the biosynthesis of anthocyanins/PAs by inhibiting *AtTT12* expression. This potential mechanism may be accounted by the protein interaction between FtMYB8 and AtTT8 which is a key member of MBW complexes (MYBs-TT8-TTG1) (Fig. 7a). This abnormal interaction may disrupt the normal formation of the MYBs-TT8-TTG1 complexes.

It has been reported that MBW complexes (AtMYBs-AtTT8/AtGL3/AtEGL3-AtTTG1) are crucial for spatiotemporal expression of *AtLBGs* and*/*or *AtTGGs* [32]. Meanwhile, Yeast two-hybrid assays showed that FtMYB8 could interact with AtTT8, not AtGL3, AtEGL3 and AtTTG1 (Fig. 7a). Thus, the expression of *EBGs* and *LBGs* was not significantly different between the WT and transgenic plants, which could be explained by the functional redundancy of MBWs. However, the functional redundancy of MBWs and this interaction between FtMYB8 and AtTT8 are not sufficient to elucidate the specific mechanism underlying the inhibition of *AtTT12* expression. Similarly, it has been reported that VvMYBC2-L1 specifically regulates the expression of the *UFGT* gene, which is regulated by VvMYBA1 [18], and LUC transient assay indicated that FtMYB8 could inhibit the transcriptional activity of *P*_*AtTT12*_ promoter (Fig. 7b). Meanwhile, TT8 interacts with TTG1 and MYBs to form MBW complexes [7]. Moreover, it has been established that a few amino acid residues of MYBs could be responsible for MBW target gene specificity [32, 34, 35]. These results allow us to hypothesize that this specific inhibitory mechanism can be attributed to the uniqueness of the DNA-binding sites of the MBW complex, although the formation of this MBW complex (FtMYB8-AtTT8-AtTTG1) has not been proven.

Because the trichomes of Arabidopsis are involved in protection against UV irradiation and insect herbivores, water regulation and temperature control, initiation of trichome formation is strictly controlled by MBW complexes, which are considered to be central to transcriptional regulation of trichome-related genes [36, 37]. However, little is known regarding the development of marginal trichomes. To date, only *TT8* and *GL3* have been proven to be essential for the induction of marginal trichomes, and the functions of these genes are partially redundant in this process [38]. In this study, a similar unique phenotype was also observed: overexpression of *FtMYB8* reduced the number of marginal trichomes in Arabidopsis (Fig. 6f), which could be attributed to the formation of the FtMYB8-AtTT8-AtTTG1 complex or decreased levels of active AtTT8 protein. These results indicated that *FtMYB8* is involved in the initiation of marginal trichome formation, although the mechanism remains unclear.

The expression and activity of MBW members is controlled strictly spatiotemporally by developmental and environmental signals because MBWs play indispensable roles in various physiological and developmental processes of plants [7]. For example, *TT8* is expressed in developing siliques, flowers, buds and 4-day-old seedlings [39]. The differential expression of inhibitors in response to developmental signals may represent different functions [18]. The role of *FtMYB8* was investigated together with analysis of the expression pattern and promoter activity of this gene at different developmental stage and under dark, UV-B, ABA and MeJA treatments, allowing us to further speculate about the physiological processes that are actually regulated in Tartary buckwheat. Our data showed that FtMYB8 strongly and weakly interacted with FtGL3 and FtTT8 (Fig. 7a), respectively, which implied that FtMYB8 can inhibits both anthocyanins/PAs accumulation and marginal trichome initiation in Tartary buckwheat may by disrupt the normal formation of the MYBs-FtGL3/FtTT8-FtTTG1 complex and/or form MBW complexes (FtMYB8-FtGL3/FtTT8-FtTTG1). The formation of these MBW complexes (FtMYB8- FtGL3/FtTT8-AtTTG1) and the function of these proteins need to be further studied. Meanwhile, qPCR results suggested that *FtMYB8* is mainly expressed in bud trichomes, roots and flowers. Moreover, this gene is expressed in bud trichomes almost throughout the developmental process, indicating that *FtMYB8* expression in bud trichomes is almost unaffected by developmental stage. These results may be associated with the role of *FtMYB8* in the initiation of marginal trichome formation. Meanwhile, *FtMYB8* expression in the roots of the true leaf stage and flowering stage is induced by specific developmental stages. These results may be associated with the inhibition of anthocyanin/PA accumulation by *FtMYB8*. Interestingly, no GUS activity was observed in the flowers of transgenic *P*_*FtMYB8*_ plants, which is not consistent with the finding that *FtMYB8* was highly expressed in the flowers of Tartary buckwheat. Therefore, we speculated that there is a potential post-transcriptional mechanism for the regulation of *FtMYB8* expression.

As previously described, the expression of *SG4-MYBs* depends on external and internal stimuli (e.g., expression of *AtMYB4* and *AtMYB7* was induced by UV-B and salt treatments, respectively) [5, 6]. Our results suggest that *FtMYB8* expression was markedly induced in the roots of Tartary buckwheat by MeJA, ABA and UV-B treatments and was repressed by dark treatment; then, the expression of this gene was downregulated in subsequent treatment periods. Considering the role of anthocyanin/PA and the function and expression pattern of FtMYB8 in Tartary buckwheat, we speculate that *FtMYB8* could also be involved in the stress response and may have a feedback regulatory mechanism.

## Conclusions

**Fig. 8:**
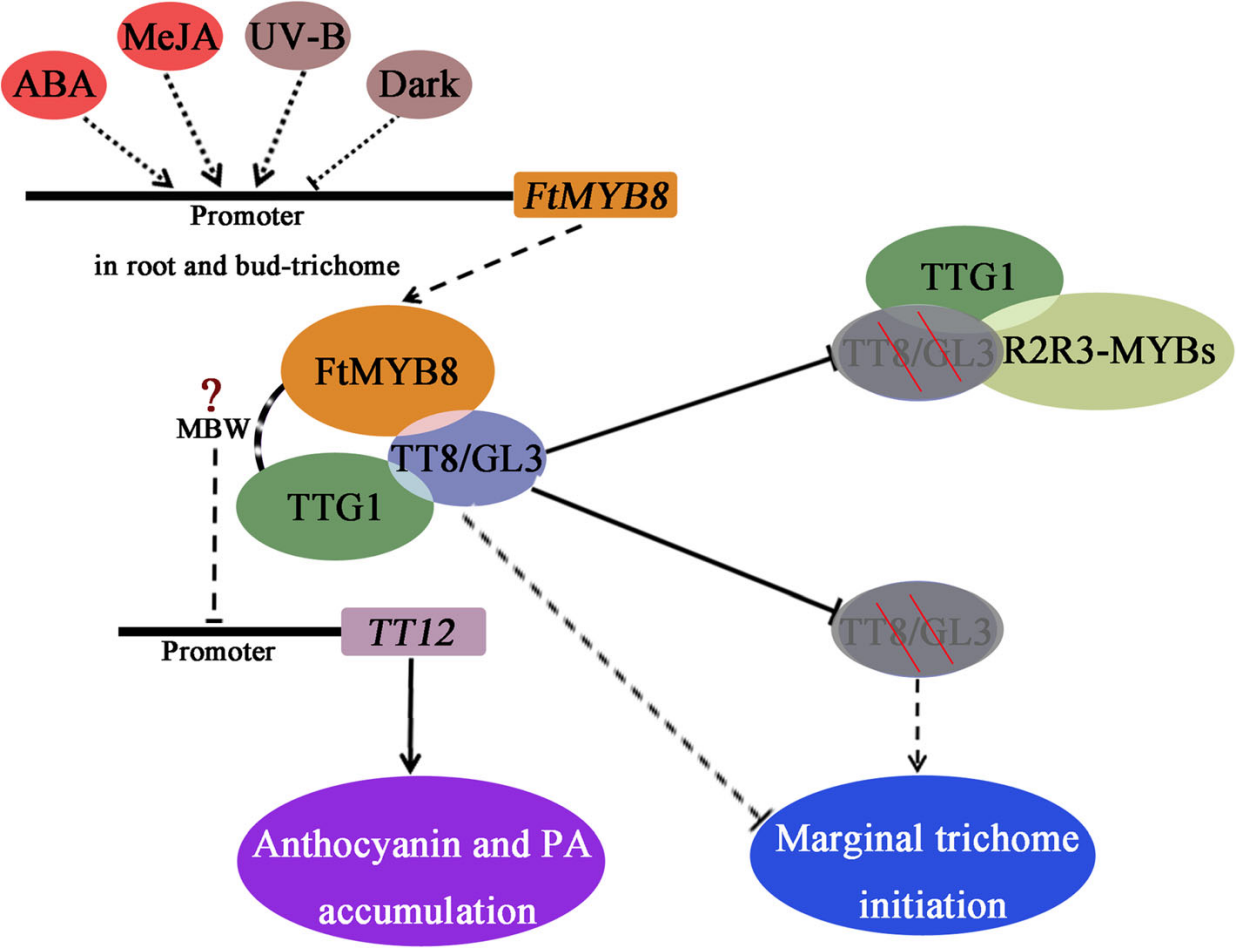
Potential working model for the function of FtMYB8. *FtMYB8* mRNA mainly accumulates in the roots during the true leaf stage and flowering stage and in bud trichomes and flowers, and the expression of this gene is enhanced by ABA, MeJA and UV-B signals and suppressed by dark treatment. FtMYB8 could interact with TT8/GL3, and this abnormal interaction may disrupt the normal formation of the MYBs-TT8/GL3-TTG1 complex. FtMYB8 may form MBW complex with TT8/GL3 and TTG1, and then, these MBW complexes may reduce the accumulation of anthocyanin/PA by downregulating *TT12* expression. FtMYB8 may also inhibit the initiation of marginal trichomes by forming MBW complexes and/or decreasing the levels of active TT8/GL3 proteins

We have proposed a potential working model for the function of FtMYB8 (Fig. 8). Our studies indicate that *FtMYB8* can play a role in the repression of anthocyanin/PA accumulation by strongly downregulate the *AtTT12* gene (not excluding FtMYB8 could also regulate other genes expression). *FtMYB8* could be involved in marginal trichome initiation. Considering the expression profile of FtMYB8 and the interaction between FtMYB8 and FtGL3/FtTT8, we propose that *FtMYB8* functions as a fine-tuning regulator of anthocyanin/PA accumulation in the roots of Tartary buckwheat and may regulate trichome distribution in the buds of Tartary buckwheat based on specific developmental stage and in response to hormone signals and environmental factors. Taken together, the results of the present study are meaningful for understanding the flavonoid regulatory mechanism of Tartary buckwheat and provide new insights into the development of marginal trichomes in *A. thaliana*.

## Methods

### Plant materials and growth conditions

Professor Anhu Wang of Xichang University gave the Tartary buckwheat accessions “Xiqiao No. 2” used in this study; Since 2013, “Xiqiao No. 2” has been introduced into the Sichuan Agricultural University, Sichuan Province, China, and grown in experimental farm. Professor Jinwen Zhang of Gansu Agricultural University gave the tobacco accessions “NC89” used in this study. Professor Yi Cai of Sichuan Agricultural University gave the *Arabidopsis thaliana* ecotype Columbia-0 (Col-0) used in this study. Seeds of Tartary buckwheat (“Xiqiao No. 2”) were sown in the farm at Sichuan Agricultural University. Buds, leaves, stems and roots of 8-, 14-, 24-, and 34-day-old plants were collected; flowers, stems, leaves and roots were collected during the flowering phase (at approximately 45 days). Seeds of Xiqiao No. 2 were germinated in a greenhouse with a 16 h photoperiod. Eight-day-old Xiqiao No. 2 seedlings were transferred into fresh Hoagland’s solution supplemented with 2 mM MeJA and 100 μM ABA. UV-B treatment was performed by transferring seedlings to a chamber subjected to UV-B illumination (302 nm, 0.1 mW/cm^2^); dark treatment was conducted using a black cover to shade the seedlings. After treatments for 0, 0.5, 1, 2, 3, 6, 10 and 16 h, whole seedlings were collected. Then, all of the above samples were stored in liquid nitrogen for subsequent experiments. Each sample contained twenty seedlings, and three technical replicates were measured for each sample.

### Isolation and characterization of the *FtMYB8* and *FtMYB8* promoters

Total RNA was isolated from various samples using the EASYspin Plant RNAiso reagent (Aidlab, China). Total RNA was used as the template for cDNA synthesis, utilizing the PrimeScript™ RT Reagent Kit (TaKaRa, Japan) following the manufacturer’s instructions. Genomic DNA was extracted from leaves utilizing the DNAquick Plant System (Tiangen, China). According to the transcriptomic database for Tartary buckwheat constructed in our laboratory (data not shown), the gene *FtMYB8* was selected, and then, the gDNA (size: 1153 bp) and cDNA (size: 729 bp) of *FtMYB8* were cloned using the *FtMYB8*F-*FtMYB8*R pair of primers (sequences reported in Additional file 1: Tables S4). Multiple sequence alignment was performed using ClustalX software, and a phylogenetic tree was generated utilizing MEGA 5.

The 5′ upstream sequence of *FtMYB8* (*P*_*FtMYB8*_, size: 2313 bp) was amplified using the P*FtMYB8*F-P*FtMYB8*R pair of primers (sequences reported in Additional file 1: Tables S4) designed based on the genomic data for Tartary buckwheat [22]. Then, the PCR product was cloned into pMD™19-T (TaKaRa, Japan) for verification by sequencing. The cis-acting elements of the *FtMYB8* promoter were analysed by using the PlantCARE database (http://bioinformatics.psb.ugent.be/webtools/plantcare/html/).

### Transcriptional assay and subcellular localization determination of FtMYB8

To investigate transcriptional activity, the CDS (coding sequence, size: 729 bp) of *FtMYB8* was amplified using the PBG*FtMYB8*F-PBG*FtMYB8*R pair of primers (sequences reported in Additional file 1: Tables S4) and then inserted into the pBridge vector to generate pBridge-*FtMYB8* using the *Sma* I and *Pst* I restriction enzymes. The pBridge-*FtbHLH2* (positive control) [40], pBridge (negative control) and pBridge-*FtMYB8* plasmids were introduced into yeast AH109 cells, and the cells were cultured in SD/−Trp-His medium. *LacZ* expression was analysed by utilizing the β-galactosidase colony lift filter assay.

The subcellular localization of FtMYB8 was determined using the roots of *FtMYB8*-overexpressing Arabidopsis plants, as described previously [41]. In brief, the roots of *FtMYB8*-overexpressing Arabidopsis plants were stained with 5 μg/ml nucleic acid dye DAPI for 5 min in the dark, washed with PBS three times, and photographed under a confocal microscope OLYMPUS FV10-MCPUS (OLYMPUS CORPORATION, Tokyo, Japan).

### Generation of transgenic Arabidopsis and tobacco plants

The CDS of *FtMYB8* lacking the stop codon (size: 726 bp) was amplified using the PYFP*FtMYB8*F-PYFP*FtMYB8*R pair of primers (sequences reported in Additional file 1: Tables S4) and then ligated into the pCHF3-YFP vector to obtain the *FtMYB8*-*YFP* fusion construct by utilizing the *Kpn* I and *Sal* I restriction enzymes. Meanwhile, a segment of the *FtMYB8* promoter (size: 2313 bp) was amplified using the PBI*PFtMYB8*F-PBI*PFtMYB8*R pair of primers (sequences reported in Additional file 1: Tables S4) and ligated into the pBI101-GUS vector by utilizing the *Hind* III and *Sal* I restriction enzymes. Then, the vectors pCHF3-*FtMYB8-YFP* and pBI101-*P*_*FtMYB8*_-*GUS* were transformed into Arabidopsis Col-0 via the Agrobacterium strain GV3101. Transgenic seeds were selected on MS medium (50 μg/mL kanamycin) and then grown in a greenhouse with 16 h light/8 h dark for 12 days. Kanamycin-resistant plants were cultured in soil-containing pots and verified by PCR analysis. Transgenic T3 homozygous lines were used as subsequent experimental materials. In addition, the recombinant vector pCHF3-*FtMYB8-YFP* was transformed into tobacco NC89 by using the Agrobacterium strain LBA4404. After rooting on selective medium (60 μg/mL kanamycin), seedlings that were approximately 7 cm high were potted in soil, identified by PCR analysis, and cultured to maturity in the greenhouse.

### Determination of flavonoid content in transgenic Arabidopsis and tobacco plants

Anthocyanins [42] and PA [43] were extracted from fresh leaves of transgenic Arabidopsis and tobacco as described previously. The anthocyanin levels were measured as described previously [44], as were the PA levels [43]. Three biological replicates were measured for each experiment.

### Yeast two-hybrid screening

According to the genome database and our transcriptome database (data not shown) combined with phylogenetic analysis results, the *FtTT8*, *FtGL3*, *FtEGL3* and *FtTTG1* have been screened out from Tartary buckwheat. Their sequences are displayed in the Additional file 1: Table S3. Yeast two hybridization assays were conducted according to previously research [45]. Briefly, the CDS of *FtMYB8* was cloned into prey vector pGADT7 by utilizing the *Sma* I and *BamH* I restriction enzymes and the CDS of *AtTT8*, *AtGL3*, *AtEGL3*, *AtTTG1*, *FtTT8*, *FtGL3*, *FtEGL3* and *FtTTG1* were cloned into bait vector pGBKT7 by utilizing the *EcoR* I and *Sal* I restriction enzymes. And then, pGADT7-*FtMYB8* prey and pGBKT7-*AtTT8*/−*AtGL3*/−*AtEGL3*/−*AtTTG1*/−*FtTT8*/−*FtGL3*/−*FtEGL3*/−*FtTTG1* bait constructs were co-transformed into yeast strain AH109 (Clontech), respectively. Co-transformation of empty prey and bait vector, empty prey and bait construct, and empty bait with prey construct were used as controls.

### Quantitative RT-PCR (qRT-PCR)

To determine the expression levels of *SG4-like-MYBs* in Tartary buckwheat, the transcriptional levels of flavonoid-related genes (*CHS*, *CHI*, *F3′H*, *FLS*, *DFR*, *ANS*, *BAN*, *TT12*, *TT19* and *AHA10*) in transgenic and WT plants, and *GUS* transcriptional levels in transgenic Arabidopsis, total RNA extraction and cDNA synthesis were performed as described previously. Three technical replicates were measured for each sample. qRT-PCR was conducted as described in previous studies [46]. *FtH3* was used as the reference gene for qRT-PCR of Tartary buckwheat; *AtActin2* was used as the reference gene for qRT-PCR of Arabidopsis; and *Ntβ-actin* was used as the reference gene for qRT-PCR of tobacco. qRT-PCR was conducted with a CFX96 Real-Time PCR Machine (Bio-Rad, U.S.A). The PCR program was: 95 °C for 20 s, 39 cycles of 95 °C for 15 s and 60 °C for 25 s. The relative gene expression data were evaluated utilizing the 2^-ΔΔCT^ method. qRT-PCR determination was conducted with three repeats. All the primers used in this study are listed in Additional file 1: Table S4.

### Dual-LUC reporter assays in tobacco leaves

Dual-LUC assays in N. benthamiana leaves were performed as previously described [47]. The AtTT12 promoter (*P*_*AtTT12*_, size: 1701 bp) was amplified using the lucPAtTT12F-lucPAtTT12R pair of primers (sequences reported in Additional file 1: Tables S4) and then ligated into the pGreenII 0800-LUC vector to obtain the reporter constructs P_AtTT12_:Luc by utilizing the *Hind* III and *BamH* I restriction enzymes. The CDS of FtMYB8 (size: 726 bp) was amplified using the FtMYB8SKF-FtMYB8SKR pair of primers (sequences reported in Additional file 1: Tables S4) and then ligated into the pGreenII 62-SK vector to obtain effector (P_35S_:FtMYB8) by utilizing the *Hind* III and *Kpn* I restriction enzymes. Transformed leaves were sprayed with and soaked in 0.1 M luciferin, after which they were placed in darkness for 6 min before luminescence examination. LUC images and quantify luminescence intensity were detected by using a charge-coupled device imaging apparatus (NightOWL II LB983 in conjunction with Indigo software). Three biological replicates were measured for each experiment, and three technical replicates were measured for each biological replicate.

### GUS histochemical assay

To study the role of the *P*_*FtMYB8*_ promoter in the growth and development process, whole 4-, 6-, 11-, 18-, 25- and 32-day-old plants and stems, leave, flowers, roots, siliques and crowded buds collected during florescence of 42-day-old plants were used for GUS staining assays.

To investigate the response of the *P*_*FtMYB8*_ promoter to environmental factors and hormones, germ-free seeds of transgenic Arabidopsis were grown on perlite with 1/2 MS liquid medium in glass bottles. When the first true leaves emerged from the sterile seedlings (at approximately 6 days), the liquid medium from the glass bottle was replaced with fresh liquid medium. Then, the glass bottles that contained the original medium remained under normal conditions, while the glass bottles that contained fresh 1/2 MS liquid medium were placed in dark, UV-B and normal conditions. Meanwhile, glass bottles that contained fresh 1/2 MS liquid medium supplemented with 10 μM ABA and 50 μM MeJA were placed under normal conditions. After treatment for 5 h, whole plants were used for GUS staining assays and RNA extraction [48].

GUS staining assays were performed as described Beeckman and Engler [49], and the transcriptional level of *GUS* in each sample was monitored using qRT-PCR. Each sample contained at least 50 seedlings, and three technical replicates were measured for each sample.

### Microscopy and histochemical analysis

Photographs of tobacco flowers and Arabidopsis germination were taken with a digital camera. Seed and seedling pigment accumulation, trichome distribution and GUS histochemical staining analyses were photographed using a stereomicroscope [10× lens in a OLYMPUS SZX2-ILLK microscope fitted with a OLYMPUS DP71 camera (OLYMPUS CORPORATION, Tokyo, JAPAN)] [50].

### Statistical analysis

Statistical analyses were conducted with SPSS software (SPSS 13.0). *P-values < 0.05 and *P-values < 0.01 were regarded as significant and extremely significant, respectively.

## Additional file

Additional file 1: **Figure S1. **Expression of *FtMYB8*during Tartary buckwheat development. **Figure S2. **Relative expression of*FtMYB8* in Tartary buckwheat seedlings under the influence of environmental factors and hormone treatments. **Figure. S3. **Transactivation assay of FtMYB8 in AH109 cells. **Figure S4.**Generation of transgenic tobacco plants. **Figure S5. **Generation of transgenic Arabidopsis plants. **Figure S6. **Subcellular localization of FtMYB8. **Figure S7. **Seed germination of WT and transgenic lines. **Table S1. **Sequence of the *PFtMYB8*promoter. **Table S2. **Predicted cis-elements of the *PFtMYB8*promoter. **Table S3. **cDNA sequences of *FtGL3*, *FtEGL3*, *FtTT8*and *FtTTG1*genes from Tartary buckwheat. **Table S4**. Sequences of all primers used in thisstudy. **Table S4**. Sequences of all primers used in thisstudy (PDF 548 kb)

## Data Availability

Data supporting the results can be found in Additional files and any other datasets used and/or analyzed during the current study is available from the corresponding author on reasonable request.

## Abbreviations

ABA: Abscisic acid
*AHA10*: Autoinhibited H^+^-ATPase isoform 10
*ANS*: Anthocyanidin synthase
*CHI*: Chalcone isomerase
*CHS*: Chalcone synthase
DAPI: 4′,6-diamidino-2-phenylindole dihydrochloride
*DFR*: Dihydroflavonol reductase
*EBGs*: Early biosynthetic genes
*F3’H*: Flavonoid 3′-hydroxylase
*FLS*: Flavonol synthase
*LBGs*: Late biosynthetic genes
MBW: MYB-bHLH-WD40
MeJA: Methyl jasmonate
PAs: Proanthocyanidins
PBS: Phosphate buffer saline
SG4: Sub-group 4
SG5: Sub-group 5
SG6: Sub-group 6
SG7: Sub-group 7
SPSS: Statistical product and service solutions
TFs: Transcription factors
*TGGs*: Third group of enzyme-encoding genes
*TT12*: Transparent testa 12
*TT19*: Transparent testa 19
*UFGT*: UDP-flavonoid glucosyltransferase
X-gal: 5-Bromo-4-chloro-3-indolyl β-D-galactopyranoside
X-α-Gal: 5-bromo-4-chloro-3-indoxyl a-D-galactoside
