# Supplementary material for: FtMYB8 from Tartary buckwheat inhibits both anthocyanin/Proanthocyanidin accumulation and marginal Trichome initiation
Source: BMC Plant Biol. 2019 Jun 18;19:263. doi: 10.1186/s12870-019-1876-x (PMC6582506; doi:10.1186/s12870-019-1876-x)
Supplement: Supplementary file 1 — Figure S1. Expression of FtMYB8during Tartary buckwheat development. Figure S2. Relative expression ofFtMYB8 in Tartary buckwheat seedlings under the influence of environmental factors and hormone treatments. Figure. S3. Transactivation assay of FtMYB8 in AH109 cells. Figure S4.Generation of transgenic tobacco plants. Figure S5. Generation of transgenic Arabidopsis plants. Figure S6. Subcellular localization of FtMYB8. Figure S7. Seed germination of WT and transgenic lines. Table S1. Sequence of the PFtMYB8promoter. Table S2. Predicted cis-elements of the PFtMYB8promoter. Table S3. cDNA sequences of FtGL3, FtEGL3, FtTT8and FtTTG1genes from Tartary buckwheat. Table S4. Sequences of all primers used in thisstudy. Table S4. Sequences of all primers used in thisstudy (PDF 548 kb) [file 12870_2019_1876_MOESM1_ESM.pdf]

FtMYB8 from Tartary buckwheat inhibits both anthocyanin/proanthocyanidin accumulation and marginal trichome initiation

Yunji Huang#, Qi Wu#, Shuang Wang, Jiaqi Shi, Qixin Dong, Panfeng Yao, Guannan Shi, Shuangxiu Xu, Renyu Deng, Chenglei Li, Hui Chen, Haixia Zhao*

Additional file

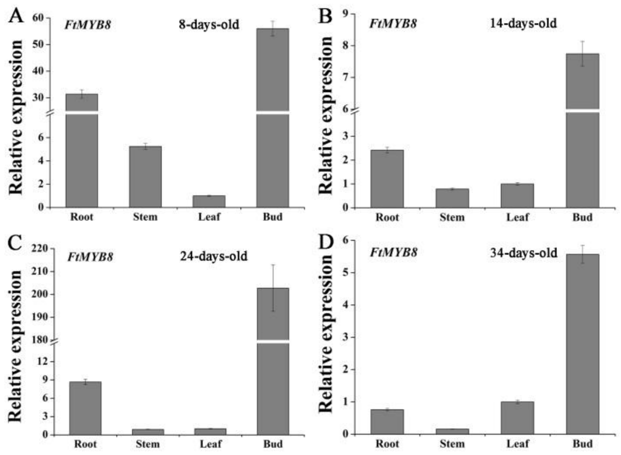

Figure S1. Expression of *FtMYB8* during Tartary buckwheat development.

The relative expression of the *FtMYB8* gene was evaluated by the $2^{-\Delta\Delta CT}$ method. *FtH3* was used as a reference gene. The accumulation of *FtMYB8* mRNA in the leaves was defined as "1" at each developmental stage. Means were calculated from three repeats, and error bars reflect ±SDs.

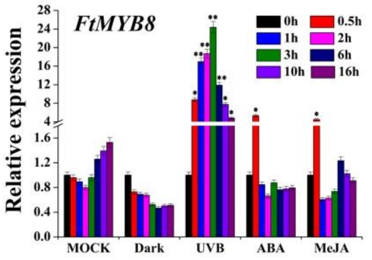

Figure S2. Relative expression of *FtMYB8* in Tartary buckwheat seedlings under the influence of environmental factors and hormone treatments.

The relative expression of the *FtMYB8* gene was evaluated by the $2^{-\Delta\Delta CT}$ method. *FtH3* was used as a reference gene. The level of *FtMYB8* mRNA at 0 h was set to "1". Means were calculated from three repeats, and error bars reflect ±SDs. $**P < 0.01$; $*P < 0.05$.

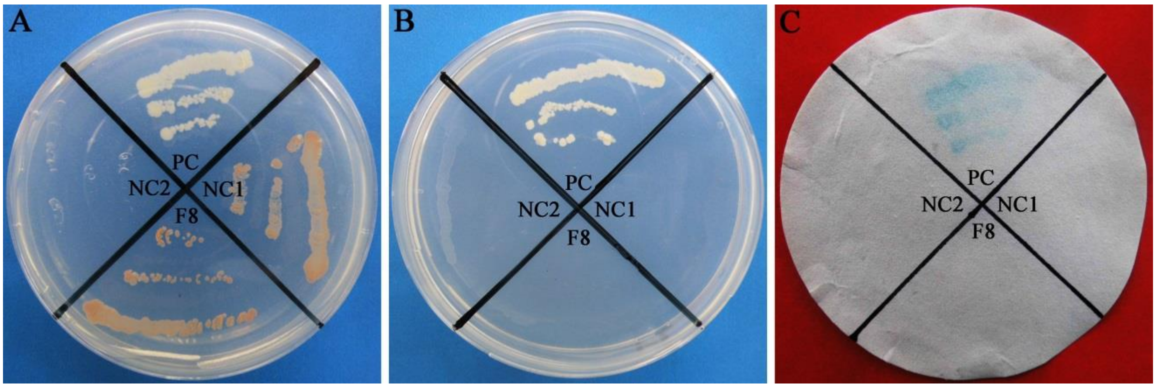

Figure S3. Transactivation assay of FtMYB8 in AH109 cells. (A-B) The transformed cells were selected on SD/-Trp and SD/-His-Trp media. (C) β-Galactosidase activity assay. PC: pBridge-*FtbHLH2*; NC1: empty plasmid; NC2: AH109 cells; F8: pBridge-*FtMYB8*.

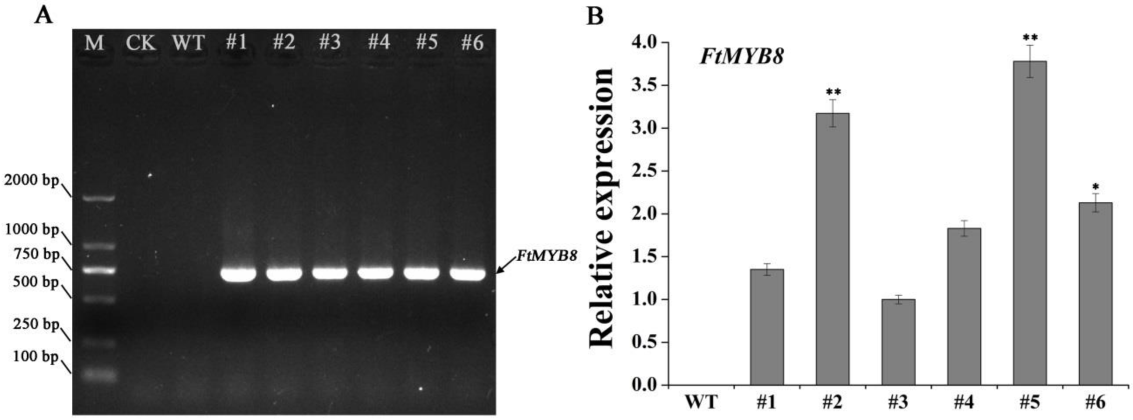

Figure S4. Generation of transgenic tobacco plants.

(A) PCR amplification of transgenic tobacco. Lane M, DNA marker 2000; lane CK, water, as a negative control; lane WT, wild-type tobacco; lanes #1 to #6, transgenic tobacco. (B) Detection of *FtMYB8* gene expression in transgenic tobacco and WT tobacco by qPCR. The tobacco *β-actin* gene served as an internal control. Error bars represent ±SDs. **P < 0.01; *P < 0.05.

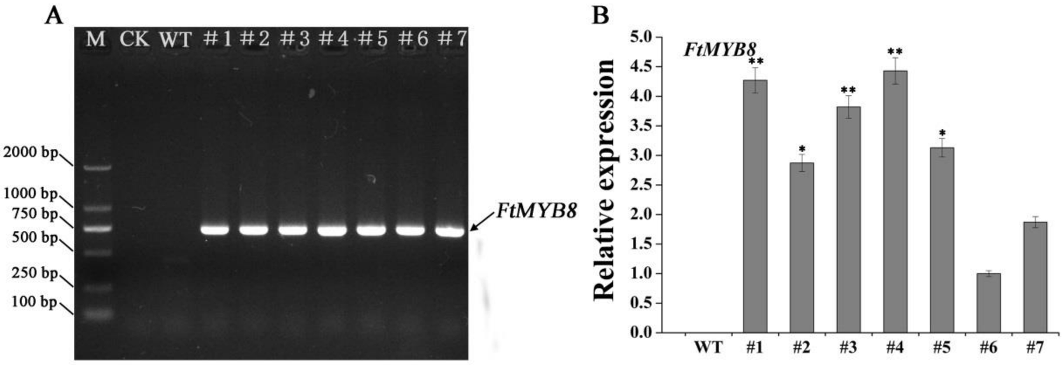

Figure S5. Generation of transgenic Arabidopsis plants.

(A) PCR amplification of transgenic Arabidopsis. Lane M, DNA marker 2000; lane WT, wild-type Arabidopsis; lane CK, water, as a negative control; lanes #1 to #7, transgenic Arabidopsis. (B) Detection of *FtMYB8* gene expression in transgenic Arabidopsis and WT Arabidopsis by qPCR. Arabidopsis *Atactin2* served as a reference gene. Error bars represent ±SDs. **P < 0.01; *P < 0.05.

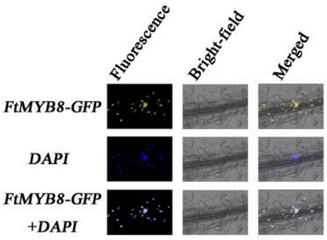

Figure S6. Subcellular localization of FtMYB8.

Root of *FtMYB8*-overexpressing Arabidopsis under a fluorescence microscope. DAPI was used as a nuclear localization marker.

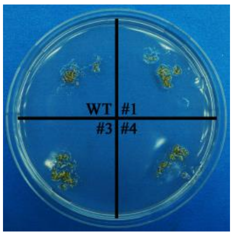

Figure S7. Seed germination of WT and transgenic lines.

WT and transgenic seeds germinated on MS medium; photos were taken after three days of germination.

Table S1. Sequence of the P_{FtMYB8} promoter

Gene	Sequence (5'→3')
P_{FtMYB8}	TAGGAAGGTATGACAGTTTGATTGGTTTATTTAGTTTTGCCTTCTAAATTTACACTTTATTCTAA<br>TTACTAGTTTACTTTAAAAACATATTGTATTTTACCTAAAAAGAACTTGATTTATCTATTACTCA<br>ATGAGTACGAACACAAAAAAAGATATGAGTGGATGATGGATCAGATGAAAATTTTAGGGGAT<br>GGATCGAGTGATGTGAGTGGTTTATCCCCGTCAATCGACCCGTTGCCGACCCTATATAAGATT<br>AAGCTAATACATCATAGCTAATTATATCCTTGTCAATTTGTAAGCTTGAATGCAAGATGCATAG<br>AGGTTATGAAAGCTTGTGGGCGTTCAATCAACATTATAGTTTAAGGTTTTTATATGTGGTAATC<br>TCTTATTGAATAACATATTTTATCAGGTGTCATTTCTATTAAATCAAAGTTAAATTAAATATTTTT<br>CTTTCAAAAAATACAAATATAAACTTATTGAAGGTAGGCTCATATTAATAACAACTCAATTTGA<br>AAGTCAATCAACAAAATCCCTAACTTGGTTGAACTTACACCAATAAAAGTTACCATTAGAAA<br>TGTGGAAATATACATTTTAAAACTACAAAAATTGGAAAAAAAAAAACCATAAAATAATCTAAA<br>AATGGATTAAGTGAAATCCAAGTTCAATAAATGTTTGTCATATATATACTTTTCTGACATTGCA<br>TTAGTATAGTTGTTAAATTAGAAATGTTATTGTCTACAATAATTTCTAACTGACAACGATGTCA<br>TCCTTTTATTTAATATTGAAAAACGTTTAAGGAAATAAAAATACTTTTTATTATTTAAAAAAAA<br>TGTTCATAGAGCATGGTAGGTTGTGCGGGTAAGTAAGAATGGCGGGATCTGCATGCGGAGGC<br>AAAAAGAAAACGACTGCTACTTAACGGTGTCATTCCCTTCACGGTAAACAACCAGTCAGCG<br>TTGTACGGTTTACGGAATCAATGCACCATTCTAACGGACTTAACCAAAATAGTTTAACAAAAC<br>CAACTAACCAAAGGTTAAATTTGTAAACGGATTTTTTTTTAAAAAATCATCAAATATTCAATTA<br>TTAACAGATTACTACATACAGTTATAAGTTATAACTTAGACAGATAACAAGTTGTGCTGTCCTT<br>ACATGTCCATCATTATTAATATTAATATAAGTTAGTTGTCTCATAAATTGGCTAAAGATATTATTT<br>TGTGGAAACAATCTAATATTAATAATGAATATTATAAACAAATTTGATGGGTTAATTTTTCTATT<br>TTCAATTGGTTTTAGTATAAAAATTTTTTTGAACATACGATGTACTTTTATCACATGATATGTTT<br>GAAATCGGACCTACCATAATTTAAAGATCTATTTTTCATTGTAAAGTCAAACTTAAAAAGTCT<br>TCACAAAGAAATGTATTTTTTTAATTATATTATATTTTTCTTTTAAACTGAGATGCTAAATAAAC<br>ATTTTTTTTCTTCATAATTTTCAGTTCTAGTTTTTAAAGCCTAAAACTTGAAAAGATAGTAAGT<br>AGTGTCCTCATCCGGAATGAGTACATGTTTTGTTTGATCATTTTCATCTAGTTTAGAATTTAAT<br>CTAGTTGGTGCCGAGCTAAAGTTTTTCCAGCTTCTAAATGTGTTTATAATCGACTTATTTAGTC<br>AAGTGAATGGGTACAAGCTAATTTTTTTTACGAGTTGATCTCAAATAGGCTGATTCATCACTA<br>CCGATTGAATATTGACTTGTCTAGTCTTTCTTAGGATTTCAAGAATGTTCTAGAACTACCCAA<br>ACTTTGGTATACTCGTGATTAGCTCGTTTGGAATCGGGTTGATGTCACAAAGTTTAAATTTAA<br>CCGAACATACAAATAAATATATTCATAATTCATCTTGTTTACTCAAGTGATCGTGTCCGAGTCT<br>GAATTGAGTTTTTCTTCGAGTTGAGCTCGGATAACTTGGTTTTTAGGTAACAAATAGAACCGA<br>TGCGAGCATTCTATTGTACGTACTTGTAGAAGTCCTATGTTAGGTAAAGTGTCGAACAAGACA<br>TTACTATGCCGTATAGTATAATAAAGAAGTTCTTCAATTCTTGGCGCCTATTTGCTTGCGTATG<br>CGTGACCCACCTAAATAGTCTCTCCTCAAACCGCCGGTCCAAGTCAACTCATCTTCGATACTC<br>TTTAAGTATTTCTTAAGAACTTGATTTTTATATATATGTGCAGCAGCTTCTGCCAGCTTTTAACG<br>CCGTAATCTATCCCGTGATCGATTTTCTTCTCTTTTGTTGTTTCTGATCGACTGAGCTTGCCGG<br>ATCAAACTCGATAACGCGA

Table S2. Predicted cis-elements of the P_{FtMYB8} promoter

**Name of the element**	**Sequence**	**Function of element**	**Number**
ABRE	GCAACGTGTC	cis-acting element involved in the abscisic acid	1

		responsiveness	
AE-box	AGAAACAA	part of a module for light response	1
ARE	AAACCA	cis-acting regulatory element essential for the anaerobic induction	6
AT-rich element	ATAGAAATCAA	binding site of AT-rich DNA binding protein (ATBP-1)	1
ATC-motif	AGTAATCT	part of a conserved DNA module involved in light responsiveness	1
Box 4	ATTAAT	part of a conserved DNA module involved in light responsiveness	4
CAAT-box	CAAT	common cis-acting element in promoter and enhancer regions	41
CCAAT-box	CAACGG	MYBHv1 binding site	1
CGTCA-motif	CGTCA	cis-acting regulatory element involved in the MeJA-responsiveness	1
GA-motif	ATAGATAA	part of a light responsive element	1
GATT-motif	CTCCTGATTAGC	part of a light responsive element	1
GT1-motif	GGTTAA	light responsive element	4
O2-site	GATGACATGG	cis-acting regulatory element involved in zein metabolism regulation	1
TATA-box	TATA	core promoter element around -30 of transcription start	60
TCT-motif	TCTTAC	part of a light responsive element	1
TGACG-motif	TGACG	cis-acting regulatory element involved in the MeJA-responsiveness	1
circadian	CAAAGATATC	cis-acting regulatory element involved in circadian control	1

Table S3. cDNA sequences of *FtGL3*, *FtEGL3*, *FtTT8* and *FtTTG1* genes from Tartary buckwheat.

Gene	cDNA sequence (5'→3')
FtGL3	ATGGAGAAAGTTTCACAAAATCTAAGAAAACAGCTTGCAATTGCTGTAAGAAGCATTCAAT<br>GGAGTTACTCAATTTTCTGGTCAATTTCCTCTTCTGAACAAGGTGTTTTGGAATGGAGTGAA<br>GGGTTTTACAATGGAGATATCAAAACAAGAAAAACTATACAAGCTGTTGAATGGGATGTAG<br>ACCAAATGAGCTTGAAAAGAAGTGAACAATTAAGAGAGCTTTATGAATCTCTCTTAACTGT

	TGAAACTAACACTCAAACTAGAAGACCTTCAGCTGCACTGATTCCTGAAGATCTTTCTGATA
	CTGAATGGTTTTATTTGGTTTGTATGTCCTTCGTCTTCAATCTCGGCCAAGGGTTACCCGGAA
	CAGCTTTACAAAATGGCGAGCCTATATGGCTATGCAATGCTCACCAAGCGGATAGCAGATTG
	TTCAGTCGCTCTCTTCTCGCTAAGAGTGCTTCAATTCAGACTGTAGTATGCTTTCCTTTCTTA
	GGAGGCGTAGTTGAGCTCGGCGCTACCGATTTGGTTTTAGAGGATCATTCATTGATCAAATA
	TGTTCAAGATTCTCTCTTGGTGACTGATTGCTCAGCACTTCCTCAGATACCCATTTCAGTAAC
	ACAATGTGAAGAGTTGAGATTGGTAGAGTGTTCGAATAACAGCTTGATTCATCTAATGGAGG
	ATACTAAATCTATGATGACTGAAAATGTAAATGTGGATCTTATAGATGATGATTTGAGTAACT
	GTGTTTATACTTCCATGACATCCAATGATTGCATATCTCAGAGCCTCGGGTATCCTGAGGATC
	TCCTTACTCTTCCGAAGATTGAGAATGTATCCGAAATTCATCCTCAAACAGCACAGGAGAAT
	ACAGAAGATAGTTGTGTTATGAGAACTGAGTTAGATGATGTTCATTACAAGAATGTTCTTGG
	TAGTATATTCAATACTAGCCACAGTTTGATCAGGGACCGTTCATATCGTAATTTTAACATGAA
	ATCATGTTTCGGTAGCTGGAGAAAAGGCGTTGGCTCGTATAGCAATCCTAGATATGGTAACT
	CGCAAAGGATCTTGAAGAAGATACTTTTTGAAGTTCCTCGGATGCATAGTGTATCCTCGTCC
	GAGAATAATGTGGATGGTGACCGGATAAAAACCGATTTGTGGAAACCCGAAGCTGATGAGC
	TTGTCTCAAACCGGATGCTTGTTCCGAGGAGGAGACGGGAGAGTGTGGATGATAGATTTTT
	CGAGTTAAAATCATTGATTCCTTCCATTGATAAGGTTGATAAGGTGTCTATACTTGATGACAC
	AATAAAGTACATGAAAGAGCTTGAGAGGAGAGTTGAAGAGCTTGAGGGAAGTCAAGATCA
	CAATGAATCCGGTGGAGGAAGAACACGTATGAAATATCAAGACGCGGCAGAAAGAACATC
	CGACAATTATAAAAACAACAAAGTCGGAATCAAGAGAAAAGCTGAGGAGGAAATTGATGA
	AGGGAAGTTAGAGAATAGTCTTGTATCAATAAGAGATAGTTCAACTAATACTAACCTATCCAT
	CAACATCATCCAAAAAGATGTCTCGATTGACATAACATGTCCTTGGAAGGAATGCTTGTTAC
	TCGAGATCATAGATGCATTAACAAATCTACGACTAGATACTCACTCGGTTCAATCCTCAAGAT
	CCGATAACCTCCTATCCATAAACATCAAAGCAAAGATCAAAGTACCGGGCGGGTTATCCGCG
	GGAATGATCAGGCAAGTGTTACAAAGAGTCGTGATAAGAAGTTGA
FtEGL3	ATGGAAACTGGGCTCCAAAACCTAAGGAAGCAGCTTGCTCTTGCTGTTAGGAGCATCCAAT
	GGAGTTACTCCATTTTCTGGTCAACTTCTCCTTCTGAACTAGGGGTACTAGAATGGGGTGAA
	GGATACTACAATGGAGATATCAAGACAAGGAAAACAATTCAAGCTGGTGAATTTGATGTAG
	ACCAGATGGGCTTGCAAAGGAGTGAGCAGTTAAGGGAGCTTTATGAGTCTCTATTAGCTGG
	TGATAGCAACCCACAAGCTAGAAGGCCATCAGCTGCACTGTCTCCTGAAGACTTGTCGAAT
	ACCGAGTGGTATTACTTGGTTTGTATGTCATTTGCCTTCAATCTTGGACAAGGGTTGCCGGG
	AAGAACTTTAGAGACCGGTCAGCCTATCTGGATCTGTAACGCCCACCAGGCAGATAGTAGT
	GTGTTTGGCCGCTCTCTGCTGGCTAAGAGTGCTTCCATTCAGACAGTGGTATGCTTTCCCTT
	TCTAGGAGGTGTCGTCGAGCTAGGTGTCACTGATATGGTTTTAGAGGATCTTCCAATGATTC
	AATATGTCCAAGCTTCTCTATTGGAGATTGATTGTCCTGCTGTTTTTAGTTATACTCGAACATC
	AGTTGTGCCATGTGAAGAAATGAGATCATTAGAGTCTCCTGATGACAGCTCTAGTGGATTTG
	GACTAATTCAACCAGTAGACGAAACTAAATTCAAGGTTGAATGTGCAAGTGGGGAGCTTAT
	AGATGATGATGACCTGAGTAACCGTGTTTATACTTCTATGACTGAAAGTGAATGTATATCACA
	GAATCTCATAAACCCTGAGAATCCCATATCTCTTTTAAATGCTCAAGAACAGGATATTCAAGA
	TGAGTTCAGTGATGATCTCCATTACCGGAATATTCTCACTAGTCTTTTCAAGAGCACTAATGA
	GTTGATCTTGGAGCCTAGTGCTTGCAATCGCGAAAACAAGTCATGTTTTGTGAGTTGGAGA
	AAAGGAGGCATAGGTTCTTGCTTTAGACATACAAATGGAAGCTCACAAAGAGTTTTAAGGA
	AAATACTATCTGAAGTTCCTCGGATGCATGATGGTTTCTCTAACCATACAATTGCAGAGAGA
	AGGAGAAGGGAGAGTGTCAATGACAGAATTTCTGCACTAAAATCACTTATCCCATCAATTG
	ATAAGGTTGACAAGGCTTCTATACTTGATGACACAATAAACTACTTGAAAAATCTCAAGAGA

	AGAGTTGAAGATCTTGAAACAAGTGAATGCAGTGAAAGAAAAACACATAAAAAGTCTCAA<br>GACATGGCAGAGAGGACATCCGACAACTACGGAAACAACAAAGATGGTGTCAAGAGAAA<br>AGCCAATGAATCTGATGAAGGGATCAGACAAAATTCTAAGAACTCTACCAAACTATCAATC<br>AGCCTAATCGAGAAGGATGTTTTAATTGAGTTAAGATGTCCATGGAAGGAATGCATATTACT<br>CGAGATCATAGATTCGCTGAGTAGTCTCAGACTAGATACACATTCGGTTGAGTCCTCTACAA<br>CTAACAACTTTCTTTCTGTTACCATCAAAGCAAAGATCAATAGATTGAGCAGTCTATCAGCA<br>GGAATGATCAAGCAGGTGCTACAGAGAGTTCTAATGAAAGCTTGA
FtTT8	ATGGCGGCACCACCGCCAGGCAACGGGCGGCTGCAGACGATGTTGCAGTCGGCGGTTCAA<br>GCCGTTCAGTGGACCTACAGTCTCTTCTGGCAATTCTGCCCCCAGCAAGGGGTTCTAGTGT<br>GGTCAGACGGATACTACAATGGAGCAATAAAGACTCGGAAAACGGTCCAGCCGATGGAGG<br>TGAGTGCCGAGGAAGCTTCCTTGCAACGCAGCCAGCAACTGCGCGAGCTTTACGACTCTCT<br>CTCAGCCGGAGACACTAACCAACCGGCAAGGCGTCCCTCTGCCGCTCTCTCACCGGAGGA<br>CTTAACGGAGACCGAGTGGTTCTACCTTATGTGTGTCTCCTTCTCTTTCCCTCCTGGTGTTGG<br>GTTACCCGGTAAGGCATACGTAAAACGACAACATGTATGGCTAACGGGCGCACACGAGGTT<br>GATAGCAAAGTCTTTTCTCGAGCTATTCTTGCTAAGAGCGCTCGGGTTCAGACGGTTGTGTG<br>TATTCCTCTGTTGGATGGAGTGCTGGAGTTGGGCTCTACTGAGAGGTTGCCGGAGGACATAT<br>CTGTTGTCCAACTCGCCAAGACGTTCTTCGTTGACCACCCTCACCACCTCCACCGCCTTCC<br>ACCGCCCAAACCGGCGCTATCTGAGCATTCCACCTCCAACCCAGCTGCCTCATCCTCCGAC<br>CACCCTGCCACCCGCTTCCACTCTCCCCCTATCCCAGCTGCCTCCATGTATCCTGCCACTGAT<br>CCTGCGACCACCTCCAATATGCACCGAGTCAACGAGGAGGCCGAGGATGACGATGAAGAA<br>GACGACAACGAATCGGATTCAGAGGCGGAAACCGGCAGAAATAGCCGATCCATGTCGGGA<br>GCAGGGCAACAAAACCCGCAAAATCCGGCTCAGTTGGGAGCTGGTGCGTCGGCAAACACG<br>GCGACTGCTGAGCCCAGTGAGCTAATGCAGCTTGAGATGTCAGAGGATATCCGCCTCGGCT<br>CACCGGAAGATGCCTCCAACAACATGGATCCCGATTTTCCGGCGACGGGTGCGGTAGCCGG<br>AATTACTATCCCGGCAAACGCGATTGTGGGTCAACAGAGAAGGGCCGACTCGTTCAGCAGG<br>GCTGAGTCATCTAGGCGTTGGCCGGTCATACAACATGATTCGTTGCATCAACAACCACCTCT<br>TCCACCGCCGCAACCACCGCCTTCCTCAGGTACCACCCCATTGGAAGAATCAGCCGCAGAG<br>GACACACACTACTCTCAAACAGTCTCAACCATTCTACAATACCAGGCCGGACGGTGGTCGG<br>AGCCCACCCCCACCACCCCAAACGGACCCAGCTACCTCAGCCTCAACACCCCACACTCCG<br>CCTTCGCAAAATGGCCGCCTCGATCCGACCACCAAGTCCACATCCCAGAAGAGCTCAACTC<br>CCAATGGCTCCTCAAATACATCCTATTCACCGTCCCATTCCTCCACACCAGTAAATCCAACG<br>ACGAAAACTCACCTAAAAGCCGAGAGCCTGGGGCTGACCAGTCCTCTCGGCTCCGAGTCA<br>AGGGCACCCCGCAAGACGAGCTCAGCGCTAACCATGTCCTAGCTGAACGAAGGCGTCGAG<br>AGAAGCTCAACGAGAGGTTCATCATACTAAGGTCGTTGGTTCCCTTTGTAACGAAAATGGAT<br>AAAGCTTCGATTTTAGGTGATACGATCGAGTACGTGAAGCAACTTCGTAGAAAAATACAAG<br>ACTTGGAAACGCGCGCAGGACAACTGGAGACCGAGCATACGCGTCTTGTTGACGCTGCTA<br>AGTCAAACAACTCAAGCGGGCTCACAGTGTCCGAGCGGGCTCGGGCTGGACTCGGGTCGA<br>GCGTGGATAAGAGGAAGATTAGGGTGGTGGAGGGGAGTAGTGGAGGTGCTAAGGCTAAGA<br>TGATTGAGTCATCACCGCCACCACTTCCACCACCATCTCAGGCAGACATGTTACAGGTGTCG<br>ATAATCGAGTGTGATGCTTTAATAGAGCTGCACTGTCCGTACAGAGAAGGGTTGCTTCTTGA<br>TGTGATGCTTGTGCTCAGGGAGTTGCGCATCGAGGTGACCGCAGTTCAATCTTCTTTGAATA<br>ATAATGGCTTCTTCGTAGCTGAAATGAGAGCTAAGGTGAAGGAGAATGAAAATGTGAAGAA<br>AGCGAGTATTGTTGAAGTGAAGAGAGCAATAAATCAGATAGTTCCGCACTCTGATGACTTCT<br>AG
FtTTG1	ATGGAGAATTCGACTCAGGAATCACACCTCCGGCATGAGAATCTGGTGACGTACGAGTCCC

	CGTACCCTCTGTACGCTATGTCGTTCGCCAGCGGCGGACACTTCCAGCGTCGGGGCGGCCG<br>AACACACGTCGCCGTCGGCAGCTTCATTGAGGAGTACAACAACAAGGTCGACATCCTCACC<br>TTCGATGACGAAACCCTAACCCTAACCCCCAATCCATCTCTTTCCTTCTCCCACCCTTATCCT<br>CCAACCAAGCTCATGTTTCGTCCCAATCCTCCCACTCCTTCTTCTTCGGCTCTCCTAGCCTCC<br>TCCGCTGACTTCCTCCGCCTCTACAGCCTCCGTAGCGGCGGCGAAGCAGTCGATCAAGTCG<br>CCGTCCTCAATAACTCCAAATCCAGCGAGTTCTGCGCCCCTCTCACCTCCTTCGACTGGAAC<br>GACCTCGTTCCGTCTCGTATCGGGACTTCAAGCATCGACACAACCTGCACGATCTGGGATAT<br>CGAGAAGGAGGCTGTGGAGACGCAGCTGATCGCGCACGACAAGGAGGTCTACGACATCGC<br>CTGGGGGGAAGCCGGCGTCTTCGCCTCCGTCTCGGCCGATGGAAGTGTTCGCATCTTCGAT<br>CTCCGTGACAAGGAGCATTCCACCATCATCTACGAGTCTCCGGAGCCGGACACCGCGCTTC<br>TCAGACTAGCCTGGAACAAGCAGGATCTCAGGTACATGGCTACGATCCTGATGGATAGCAA<br>CAAAGTGGTAATACTCGACATCCGCGTTCCTACTATGCCTGTTGCGGAGCTTGAGAGACATG<br>GCGCTAGCGTCAACGCCATAGCCTGGGCTCCTCAGAGCTCCAGGCATATCTGCTCTGCCGG<br>AGATGACGCCCAGGCACTCATCTGGGAGCTTCCTACCGTGGCTGGCCCCGCTGGAATCGAT<br>CCTCTATCTTGTTACTCTGCTGCCTCCGAGATTAATCAGTTGCAGTGGTCTGCAACGCAGCC<br>TGACTGGATTTCCATCGCCTTTGCCAATAAATTGCAGATGCTGAAAGTTTAA

Table S4. Sequences of all primers used in this study

Primer name	Forward primer (5'→3')	Reverse primer (5'→3')
FtMYB8	ATGAGGAAGCCGTGTTGCGAGA	TCATCTGAATAGGTTAAGAGTAGGA
PFtMYB8	TAGGAAGGTATGACAGTTTGATTG	TGCTTGGACCAAGCTCCCTTG
PBG-FtMYB8	TGACTGTATCGCCGGAATTCATGAG<br>GAAGCCGTGTTGCGA	TAGCTTGGCTGCAGGTCGACTCATCT<br>GAATAGGTTAAGAGTAG
PYFP-FtMYB8	GACGAGCTCGGTACCATGAGGAAG<br>CCGTGTTGCGAG	GCTCACCATGTCGACTCTGAATAGGTT<br>AAGAGTAGG
PBI-PFtMYB8	TGATTACGCCAAGCTTTAGGAAGG<br>TATGACAGTTTGATTG	ATCCTCTAGAGTCGACTCGCGTTATCG<br>AGTTTGATCCGGC
PGAD-FtMYB8	TGAATTCCACCCGGGT<br>ATGAGGAAGCCGTGTTGCGAGA	GAGCTCGATGGATCC<br>TCATCTGAATAGGTTAAGAGTAG
PGBK-AtTT8	ATGGAGGCCGAATTCATGGATGAA<br>TCAAGTATTATTC	CCGCTGCAGGTCGACCTATAGATTAGT<br>ATCATGTATT
PGBK-AtGL3	ATGGAGGCCGAATTCATGGCTACC<br>GGACAAAACAG	CCGCTGCAGGTCGACTCAACAGATCC<br>ATGCAACCC
PGBK-AtEGL3	ATGGAGGCCGAATTCATGGCAACC<br>GGAGAAAACAG	CCGCTGCAGGTCGACTTAACATATCCA<br>TGCAACCC
PGBK-AtTTG1	ATGGAGGCCGAATTCATGGATAATT	CCGCTGCAGGTCGACTCAAACTCTAA

	CAGCTCCAGA	GGAGCTGCA
PGBK-FtTT8	ATGGAGGCCGAATTCATGGCGGCACCACCGCCAGG	CCGCTGCAGGTCGACCTAGAAGTCATCAGAGTGCG
PGBK-FtGL3	ATGGAGGCCGAATTCATGGAGAAAGTTTCACAAAATC	CCGCTGCAGGTCGACTCAACTTCTTATCACGACTCTT
PGBK-FtEGL3	ATGGAGGCCGAATTCATGGAAACTGGGCTCCAAAA	CCGCTGCAGGTCGACTCAAGCTTTCATTAGAACTCTC
PGBK-FtTTG1	ATGGAGGCCGAATTCATGGAGAATTCGACTCAGGA	CCGCTGCAGGTCGACTTAAACTTTCAGCATCTGCA
FtH3	AATTCGCAAGTACCAGAAG	CCAACAAGGTATGCCTCAGC
qFtMYB8	TTGATTGCGGGGAGGTTACC	TCGGTTATCTGCGAGACGTG
qFtPinG0505906200.01	GCGTTCTATTCCTAAAGCTGCC	CTTCCCGCAATCAAAGACCA
qFtPinG0606379100.01	AAGCATGGAGAGGGGTGTTG	TCCGTTCTTCCTGGTAGCCT
qFtPinG0100390100.01	TCACAAACTCCTCGGGCTCA	TGGCATGGCAATGGTTAGATC
AtActin2	CTGGAATGGTGAAGGCTGGTT	CGATTGGATACTTCAGAGTGAGGAT
Ntβ-actin	TATCCAGAAAGAGTCAACCCGTCACCT	TAGACAAAGCACATCACGACCACAACC
GUS	ATGCGGACTTACGTGGCAAAGGATTCGA	GCCGACAGCAGCAGTTTCATCAATCACC
NtCHS	ACAAGACATAGTGGTGGTTGAAGTGCC	GAGCCCGAGTAGCTTAGTGAGTTGGTAG
NtCHI	GTGCTCTTCCTTTTCTCGCCGC	ATTTTCTGCCACCTTCTCTGAGTATTG
NtF3’H	GGTGGTTGTGGCGTCTGCGTCG	AAGCCTTGGCAGAGAAAAGATGAACAGA
NtFLS	ACAGGCAATAGCGTCAATAACAAAATG	GCTAGCATCCGATATGAGTTTCACTACA
NtDFR	GCTAAAGCAAACACAGTGAAGAGGC	CATCCATCCTGTCATCTTCTTAGCATA
NtANS	AGTATGCTAATGACCAACCCTCTG	TTGTAACCCAGGCACCATATTGTG
AtCHS	ACGGACATTTGAGGGAAGTTGG	AGGGTGGGCTATCCAGAAGAGG

AtCHI	GCGGTTCTGGAATCTATCATCG	TCGTCCTTGTTCTTCATCATTAGC
AtF3'H	TCGTGGTCGCCGCTTCTAA	ATCGGTGTCCGTAAGGTGCA
AtFLS	ACCGTTTGCTTTCAAGGATTACAGTTAC	AAATGATCACCGATTTTTTTCCGTG
AtDFR	GCCAAACGCCAAGACGCTA	CATTCACTGTCGGCTTTATCACTTC
AtANS	AGGTTAGGATTTCTTGGGCTGTG	CCGTGGAGGAAACTTAGCCG
AtBAN	GCACGGGAAACTTAGCCTCT	GATCGGAGTTGCGACATGGA
AtTT12	TGGTACTCGGGTCCAATCCT	CCAAGCAAGCCGAAATCCAG
AtTT19	CATCTTCTTCGTCAGCCATTTGGT	CGTCACATTTCTCGCCTAACCT
AtAHA10	CCGAGGATTTGGACAAGCCA	GACCTAGACTTTGACTATTAGC
